# A selective ATP-competitive sphingosine kinase inhibitor demonstrates anti-cancer properties

**DOI:** 10.18632/oncotarget.3178

**Published:** 2015-03-11

**Authors:** Melissa R. Pitman, Jason A. Powell, Carl Coolen, Paul A.B. Moretti, Julia R. Zebol, Duyen H. Pham, John W. Finnie, Anthony S. Don, Lisa M. Ebert, Claudine S. Bonder, Briony L. Gliddon, Stuart M. Pitson

**Affiliations:** ^1^ Centre for Cancer Biology, University of South Australia and SA Pathology, Adelaide, SA 5000, Australia; ^2^ School of Molecular and Biomedical Science, University of Adelaide, SA 5005, Australia; ^3^ School of Medicine, University of Adelaide, SA 5005, Australia; ^4^ School of Veterinary Science, University of Adelaide, SA 5005, Australia; ^5^ SA Pathology, Hanson Institute Centre for Neurological Diseases, Adelaide, SA 5000, Australia; ^6^ Prince of Wales Clinical School, University of New South Wales, Sydney, NSW 2052, Australia

**Keywords:** Apoptosis, *in silico* docking, molecular modeling, small molecule inhibitor, sphingosine kinase

## Abstract

The dynamic balance of cellular sphingolipids, the sphingolipid rheostat, is an important determinant of cell fate, and is commonly deregulated in cancer. Sphingosine 1-phosphate is a signaling molecule with anti-apoptotic, pro-proliferative and pro-angiogenic effects, while conversely, ceramide and sphingosine are pro-apoptotic. The sphingosine kinases (SKs) are key regulators of this sphingolipid rheostat, and are attractive targets for anti-cancer therapy. Here we report a first-in-class ATP-binding site-directed small molecule SK inhibitor, MP-A08, discovered using an approach of structural homology modelling of the ATP-binding site of SK1 and *in silico* docking with small molecule libraries. MP-A08 is a highly selective ATP competitive SK inhibitor that targets both SK1 and SK2. MP-A08 blocks pro-proliferative signalling pathways, induces mitochondrial-associated apoptosis in a SK-dependent manner, and reduces the growth of human lung adenocarcinoma tumours in a mouse xenograft model by both inducing tumour cell apoptosis and inhibiting tumour angiogenesis. Thus, this selective ATP competitive SK inhibitor provides a promising candidate for potential development as an anti-cancer therapy, and also, due to its different mode of inhibition to other known SK inhibitors, both validates the SKs as targets for anti-cancer therapy, and represents an important experimental tool to study these enzymes.

## INTRODUCTION

A number of sphingolipids, including ceramide, sphingosine and sphingosine 1-phosphate (S1P), are important signaling molecules controlling a diverse array of important cell processes [1]. S1P, in particular, has diverse cell signaling roles through its actions as both a ligand for a family of five S1P-specific G protein-coupled receptors (named S1P_1–5_), as well as a modulator of a range of intracellular proteins [2–4]. S1P receptor-mediated signaling most notably plays significant roles in immune cell trafficking and vascular integrity, while S1P in general confers pro-proliferative, pro-survival signaling [5]. Sphingosine and many ceramide species, however, are pro-apoptotic, modulating the activity of a range of enzymes involved in the control of cell survival [5]. Thus, the balance between the cellular levels of S1P and ceramide/sphingosine, the so-called sphingolipid rheostat, appears an important regulator of cell fate.

The cellular levels of the sphingolipids are controlled by an array of bidirectional metabolic pathways that are subject to complex spatial and temporal regulation [1, 6]. Some of the most important regulators of this system are the sphingosine kinases (SKs), which, through their action of phosphorylating sphingosine to generate S1P, play a vital role in controlling the sphingolipid rheostat [1], and therefore, cell fate. Two SKs exist in mammals; SK1 and SK2, which catalyze the same reaction and share a high degree of sequence similarity. The two SKs share some redundant and related roles, but also appear to possess some different functions, probably due to their different subcellular localizations, with SK1 predominantly localized to the cytoplasm while SK2 is mainly localized at the nucleus and other organelles [7].

The SKs have been widely implicated in carcinogenesis. SK1 expression is elevated in a wide array of human solid cancers, with higher levels of SK1 correlating with the severity of malignancy and shorter patient survival [8]. Similarly, SK2 was recently found to be elevated in human non-small cell lung cancer, with high expression levels correlated with poor patient survival [9]. Furthermore, a large number of studies have shown that targeting SKs has considerable potential as an anti-cancer strategy. For example, RNAi-mediated knockdown or inhibition of SK1 and SK2 has been widely demonstrated to induce apoptosis and enhance sensitivity to chemo- or radiation therapy of many different cancer cells [10, 11]. Similarly, genetic ablation of SK1 and SK2 in mice was found to reduce tumor growth *in vivo* in numerous cancer models [12–19]. This body of evidence has secured the SKs as promising therapeutic targets in cancer and has driven drug development to target the enzymes in a range of cancer models [10, 11].

Initial SK inhibitor development used molecules derived from sphingosine including L-*threo*-dihydrosphingosine, *N,N*-dimethylsphingosine and *N,N,N*-trimethylsphingosine. These compounds, however, have low specificity, with several important off-targets identified, such as protein kinase C, ceramide synthase and the 14–3-3 pro-survival protein [11, 20]. Other inhibitors developed to more selectively target the sphingosine-binding pockets of SK1 and SK2 have been successful in blocking cancer cell growth and reducing tumor burden in animal models [2, 11]. Again, however, these second generation SK inhibitors also appear to have numerous off-targets, with the most commonly employed SK inhibitor in the last decade, SKI-II [21] recently shown to inhibit dihydroceramide desaturase [22] and also enhance signalling via the Nrf2 transcription factor in a SK-independent manner [23]. Similarly, the most commonly used SK2-selective inhibitor ABC294640 has been shown to also act as a direct antagonist of the estrogen receptor [24].

Notably, some high affinity sphingosine-competitive SK1 inhibitors developed recently have been controversial. Despite showing potent SK1 inhibition *in vitro* and decreases in S1P in cells, these inhibitors failed to induce apoptosis or show anti-neoplastic properties *in vivo* [25–27]. This has lead to the groups that developed these reagents to reach the contentious conclusion that SK activity is not required for tumor cell viability [26], despite the large body of evidence to the contrary. Notably, unlike other SK inhibitors or SK knockdown, these recent inhibitors failed to enhance cellular ceramide levels at low concentrations where SK1 was inhibited [25–27]. This suggests the similarity of these molecules to sphingosine may result in off-target inhibition of ceramide synthases which blocks ceramide generation and associated pro-apoptotic signaling.

Here we describe the discovery and characterization of a novel SK inhibitor, MP-A08, using a structure-based approach to target the ATP-binding pocket of SK1. Via this approach we both exploit the known divergence of the SK ATP-binding site from other kinases [28] and also overcome common off-target effects of sphingosine-like molecules. Characterization of MP-A08 demonstrated its high selectivity to SK1 and SK2 over other kinases, and importantly revealed its anti-neoplastic effects against a panel of cancer cell lines *in vitro* and also in human lung tumor xenografts in mice.

## RESULTS

### Modeling and validation of the ATP-binding pocket of SK1

**Figure 1:**
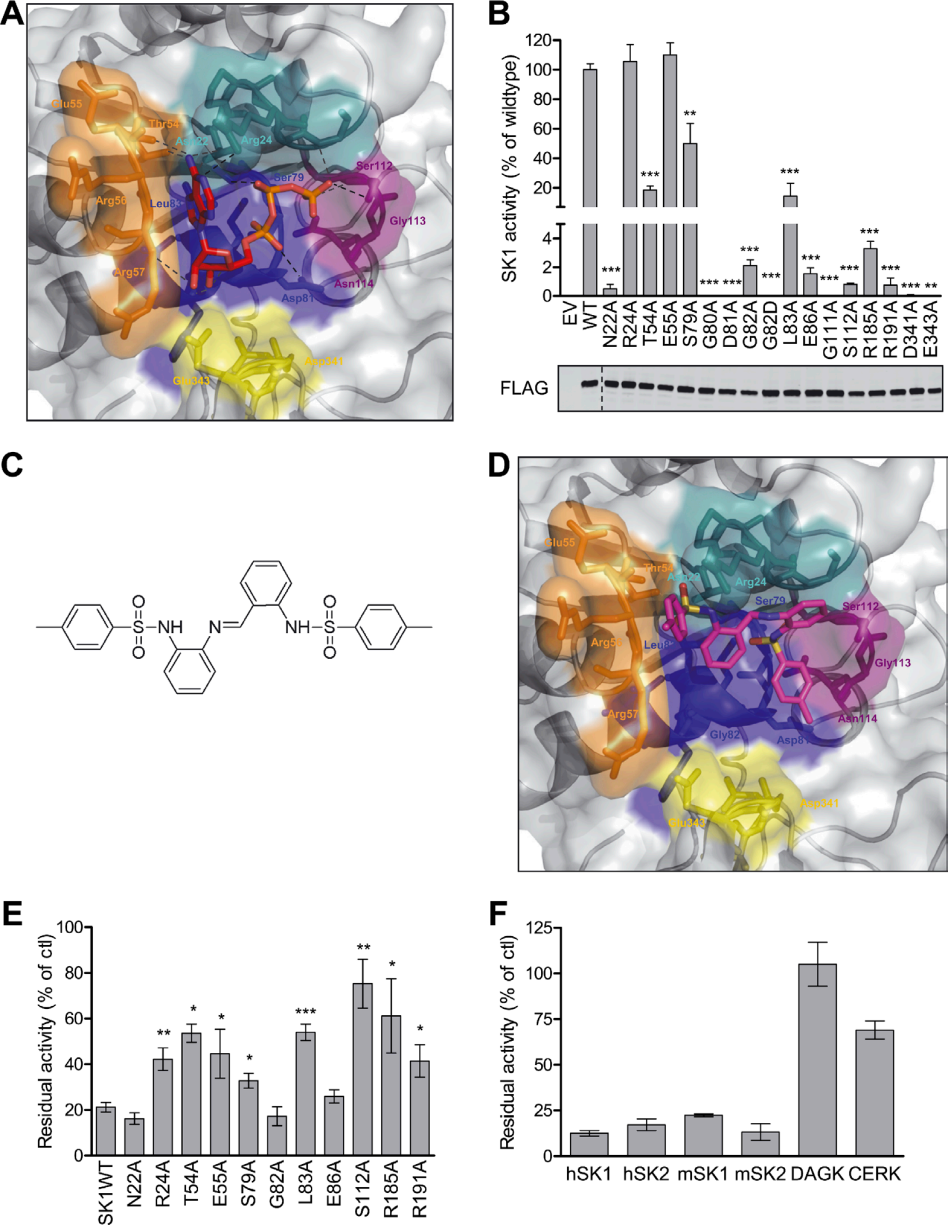
Structural modeling, analysis and virtual screening of the ATP-binding pocket of SK1 **(A)** The predicted structure of the ATP-binding pocket of SK1 represented in surface view with ATP docked into the pocket (sticks). Each of the regions highly conserved in all SKs (see Supplementary Figure 1) that comprise the ATP-binding pocket are colored separately; Motif 1 (^21^LNPRGG^26^) in teal, Motif 2 (^54^TERR^57^) in orange, Motif 3 (^79^SGDGLMHE^86^) in blue, Motif 4 (^110^GSGN^114^) in purple, and Motif 5 (^340^VDGE^343^) in yellow. The atoms in ATP are colored according to chemical elements; oxygen in red, and nitrogen in blue. Predicted hydrogen bonding is depicted by black dashes. **(B)** Mutagenesis of SK1 at residues predicted to be important in ATP binding and assessment of residual SK1 activity (upper panel) confirmed the validity of the SK1 ATP-binding site model. SK1 activities were determined after overexpression in HEK293T cells, and presented as % activity compared to wildtype SK1 (WT). Empty vector (EV) transfected cells show negligible contribution from endogenous SK to the activities displayed. The lower panel shows similar expression levels of all FLAG-tagged SK1 variants, but activities were adjusted for slight variations in expression. Data shown are mean ± SD, *n* = 4). Significance compared to WT was determined by student *t*-test (*p < 0.05, **p < 0.01 and ***p < 0.001). **(C)** The chemical structure of MP-A08. **(D)** The ATP binding pocket of the SK1 model with MP-A08 docked. Coloring is the same as for Figure 1A, with yellow atoms representing sulphur. **(E)** Effect of MP-A08 (250 μM) on the activity of SK1 variants harboring mutations in the ATP-binding site. Data are represented as % activity compared to vehicle control. All data shown are mean ± SD (*n* = 4), significance compared to SK1WT was determined by student *t*-test (*p < 0.05, **p < 0.01 and ***p < 0.001). **(F)** Selectivity of MP-A08 (0.1 mg/ml, 192 μM) was initially assessed against human SK1, SK2, DAGK and CERK, and murine SK1 and SK2. Data are represented as % activity compared to vehicle control. All data shown are mean ± SD (*n* = 4).

Until recently there was no structural information available for SK1. Therefore, we employed homology modeling to predict the structure of the ATP-binding pocket of SK1 using the solved structures of two bacterial lipid kinases, DgkB [29] and YegS [30] that, while possessing little overall sequence similarity to SK1, do show some sequence similarity with residues proposed to contribute to ATP binding in SK1 [1, 28, 31] (Supplementary Figure 1). In the model (Figure 1A), residues from all five regions highly conserved in the SKs (Motifs 1–5; Supplementary Figure 1) were involved in forming the ATP-binding pocket of SK1, with the ^79^SGDGLMHE^86^ motif forming the centre of the pocket. To identify potential residues most important for ATP binding we next computationally docked ATP into the predicted SK1 ATP-binding pocket (Figure 1A). The docked ATP was predicted to form hydrogen bonds with multiple residues in Motifs 1 to 4 (Asn22, Thr54, Gly80, Asp81, Gly82, Leu83, Glu86 and Ser112) (Figure 1A). Additionally, Asp341 and Glu343 from Motif 5 were predicted to coordinate a magnesium ion, as inferred from similarity to the DgkB structure [29].

To validate the modeling and ATP docking we then carried out alanine mutagenesis of the residues predicted to contribute to the ATP-binding pocket. Mutagenesis of all the SK1 residues predicted to form hydrogen-bonds with ATP either abolished or substantially reduced enzyme activity, while mutation of other residues in this region not predicted to directly interact with ATP (Arg24, Glu55 and Ser79) generally had less effect (Figure 1B). Mutation of Asp341 and Glu343 abolished SK1 activity, consistent with their proposed role in the coordination of the magnesium ion cofactor. Together, these findings supported the accuracy of the model. Notably, the recent availability of the SK1 crystal structure [32] also enabled us to retrospectively assess the validity of our model. Structural alignment of the ATP-binding pocket residues of our SK1 ATP-binding site model with those of the ADP-bound SK1 crystal structure (3VZD) showed very close alignment (RMSD = 1.1366 Å), further confirming the validity of our model (Supplementary Figure 2). Interestingly, the main differences between our model and the SK1 structure were in Arg185 and Arg191, which were excluded from the model because the sequence identity in that region was below the required threshold for homology modeling (< 30%). These residues contribute to binding of the β-phosphate of ADP in the crystal structure and were confirmed to contribute to ATP binding as alanine mutations of Arg185 and Arg191 substantially reduced SK1 activity (Figure 1B).

### Virtual screening identifies MP-A08 as a novel SK-selective inhibitor

The validated model of the ATP-binding pocket of SK1 was then used in a virtual screen to identify novel inhibitors of SK1. Libraries composed of 120,000 compounds were docked into the ATP-binding pockets of the SK1 model and DgkB structure using a two-step screening approach. Candidate compounds were chosen that displayed preferential docking scores and orientation for the ATP-binding pocket of SK1 over that of DgkB. Physical screening of the top candidate compounds was then performed to examine their ability to inhibit the activity of purified recombinant SK1 *in vitro*. From this screening we identified several novel hit molecules (data not shown) however due to superior inhibition and solubility 4-methyl-N-[2-[[2-[(4-methylphenyl)sulfonylamino]phenyl]iminomethyl]phenyl]benzenesulfonamide (henceforth referred to as MP-A08) (Figure 1C) was chosen for further investigation as an inhibitor of SK1. The molecule contains two benzenesulfonamide groups joined by a central benzylideneaniline group.

MP-A08 docked into the ATP-binding pocket of SK1 in close association with conserved Motifs 1–3, and was predicted to form close associations with Asn22, Arg24, Thr54, Ser79, Gly80, Asp81, Gly82, Leu83 and Ser112 (Figure 1D). To confirm this orientation of MP- A08 binding we next assessed its ability to inhibit the ATP-binding pocket mutants of SK1 (Figure 1E). MP-A08 inhibition of SK1 was reduced by around three-fold by the T54A, L83A, R185A and S112A mutations and approximately two-fold by the S79A, R24A and R191A mutations. Interestingly, the R24A mutation did not affect SK1 activity (Figure 1B) but reduced MP-A08 inhibition (Figure 1E), which was in agreement with the docking where the Arg24 side-chain was overarching the central phenyl rings of MP-A08 (Figure 1D). These findings confirm that MP-A08 is an inhibitor targeting the ATP-binding pocket of SK1. Together with the docking it also suggests that a subset of the residues that bind the adenosine component of ATP, namely Arg24, Thr54, Glu55 and Leu83, accommodate the amine group from the benzene-sulfonamide and the central imine in MP-A08. Additionally, the polar side-chains of Ser79 and Ser112 and the positively charged Arg185 and Arg191 which coordinate the negatively charged phosphates of ATP, accommodate the bulky phenyl rings and sulfonyl group in MP-A08. A lack of activity precluded us from testing the affect of mutation at Asp81 on MP-A08 binding; however, it is predicted that the acidic side-chain of this residue would also contribute to MP-A08 binding.

To assess its selectivity, MP-A08 was tested against purified recombinant human SK1 and SK2, with both enzymes being similarly inhibited (Figure 1F). Notably, unlike some other recently developed SK inhibitors [26], comparable inhibition of murine SK1 and SK2 was also observed (Figure 1F). We also examined the inhibitory activity of MP-A08 against the related human lipid kinases, diacylglycerol kinase (DAGK) and ceramide kinase (CERK), which both show considerable polypeptide sequence similarity to the SKs in most of the conserved regions involved in forming the ATP-binding pocket [31] (Motifs 1–5). Notably, MP-A08 showed no inhibition of human DAGK, and only weakly inhibited CERK when employed at 192 μM (Figure 1F). Since SKI-II was recently shown to inhibit dihydroceramide desaturase [22] we also tested whether MP-A08 had any effects on this enzyme. While, consistent with this previous study, dihydroceramide desaturase activity was substantially blocked by 10 μM SKI-II, no effects were observed by 20 μM MP-A08 (data not shown). To further assess specificity, we next tested MP-A08 against a panel of 140 human protein kinases. Consistent with the structural divergence of the ATP-binding pocket of SK1 from that of the protein kinases, initial screening showed very few protein kinases were affected by 25 μM MP- A08, and those that were only displayed modest inhibition (Supplementary Table 1). Further analysis using very high concentrations of MP-A08 (250 μM) against seven protein kinases that were inhibited by more than 30% in the initial screen, failed to show a dose-dependent trend for six of the enzymes. Only testis-specific serine kinase 1 (TSSK1) was modestly inhibited (by 57%) at this very high concentration of MP-A08 (Supplementary Table 1).

**Figure 2:**
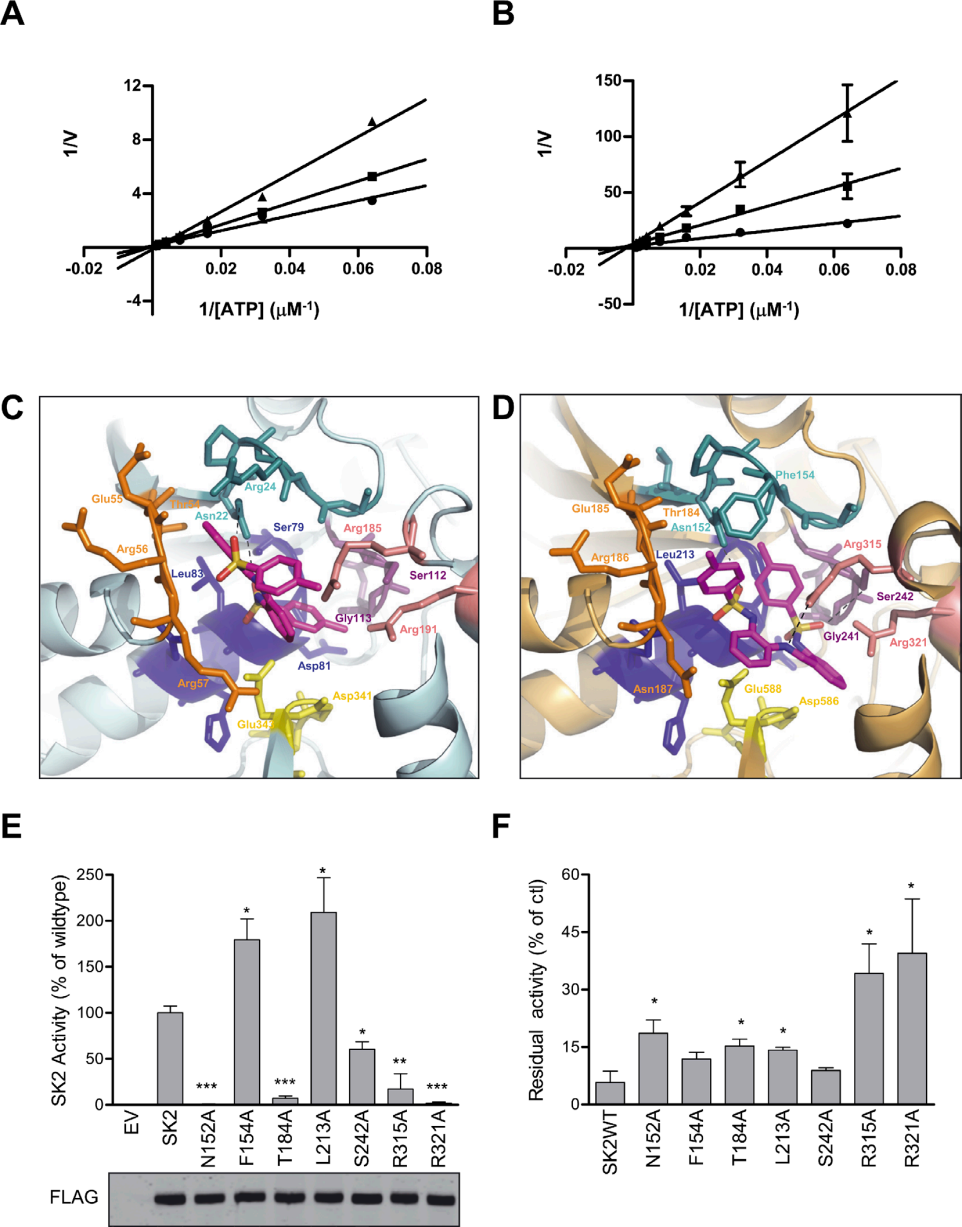
MP-A08 is a novel ATP-competitive inhibitor for SK1 and SK2 Lineweaver–Burke plots showing inhibition kinetics MP-A08 against recombinant SK1 **(A)** and SK2 **(B)** with varying ATP concentration. Data show MP-A08 employed at 50 μM (▲) or 25 μM (■), or with vehicle control (●), and are mean ± SD from four independent experiments. **(C)** The ATP-binding pocket of the recently solved SK1 crystal structure (3VZD) with MP-A08 docked. Each of the regions highly conserved in all SKs (see Supplementary Figure 1) that comprise the ATP-binding pocket are colored in the same scheme as in Figure 1A. SK1^Arg185^ and SK1^Arg191^ are colored in light pink. The atoms in MP-A08 are colored according to chemical elements; oxygen in red, nitrogen in blue, and sulphur in yellow. **(D)** The predicted SK2 model is represented with MP-A08 docked. Each of the conserved motifs that comprise the ATP-binding pocket are colored as in C. SK2^Arg315^ and SK2^Arg321^ are colored in light pink. **(E)** Assessment of residual SK2 activity in the ATP-binding pocket mutants of residues predicted to be important for SK2 binding to MP-A08. SK2 activities were determined after overexpression in HEK293T cells, and represented as % activity compared to wildtype SK2 (WT). Empty vector (EV) transfected cells show negligible contribution from endogenous SK to the activities displayed. The lower panel shows similar expression levels of all SK2 variants, but all activities were adjusted for slight variations in expression. All data shown are mean ± SD (*n* = 4). **(F)** Effect of MP-A08 (250 μM) on the activity of SK2 variants harboring mutations in the ATP-binding site. Values are represented as % activity compared to vehicle control, mean ± SD (*n* = 4). Significance from SK2WT was determined by student *t*-test (*p < 0.05, **p < 0.01 and ***p < 0.001)

Inhibition kinetics confirmed that MP-A08 was an ATP-competitive inhibitor of human SK1 and SK2 (Figure 2A–2B). Somewhat surprisingly, MP-A08 was a higher affinity inhibitor of SK2 than SK1, with *K*_i_ values of 6.9 ± 0.8 μM and 27 ± 3 μM, respectively. In order to analyze these differences in binding to SK1 and SK2 we produced a homology model of SK2 using the recent SK1 crystal structure, and then docked MP-A08 into the SK1 crystal structure (Figure 2C) and the predicted SK2 ATP-binding pocket (Figure 2D). Comparison of the SK1 crystal structure and the SK2 model revealed a highly conserved ATP pocket with the exception of the substitutions at SK2^Phe154^/SK1^Arg24^ and SK2^Asn187^/SK1^Arg57^ (Figure 2C–2D, Supplementary Figure 3A–3B). In the SK1 structure Arg24 and Thr54 appear to coordinate one of the central phenyl rings of MP-A08 and shield the amine groups from the basic side-chains of Arg24 and Arg57. The second central phenyl ring likely shields the internal amine group from the Arg185 and Arg191 side-chains, pointing the sulfonyl groups towards Asn22, Ser79 and Leu83 in SK1 (Figure 2C). One methyl substituted ring points out towards the outside of the pocket and the other is orientated towards the internal Ser112 and the Arg185 and Arg191 side-chains in SK1. The predicted orientation of MP-A08 in the ATP-binding pocket of SK2 is altered due to the substituted residues at Phe154 and Asn187 (Arg24 and Arg57 in SK1, respectively). The bulky aromatic side-chain of Phe154 and the smaller basic side-chain of Asn187 alter both the size and charge of the ATP pocket in SK2 (Figure 2D and Supplementary Figure 3A–3B). The terminal methylphenyl rings point towards the side-chain of Thr184 and Asn152. Compared to the SK1 pocket, the central phenyl rings of MP-A08 are shifted towards the bottom of the SK2 pocket, coordinated by Arg315 and Arg321. The sulfonyl groups are tethered via hydrogen bonding to Asn152 and Ser242 side-chains.

To validate the SK2 model we tested alanine mutants of the SK2 ATP pocket predicted to contribute to MP-A08 binding. As was found with the corresponding residues in SK1, SK2 activity was significantly reduced with alanine mutations at Asn152, Thr184, Ser242, Arg315 and Arg321 (Figure 2E). Conversely, alanine mutations of either Phe154 or Leu213 displayed a two-fold increase in SK2 activity (Figure 2E), due to the removal of the bulky side-chains. These residues are involved in binding the adenosine of ATP, therefore removal of either of these side-chains appears to reduce steric hindrance in this region of the pocket. We next assessed the SK2 mutants for MP-A08 binding. In agreement with the docking we found that alanine mutations at Asn152, Thr184 and Leu213 gave some reduction in SK2 inhibition by MP-A08, while mutations at Arg315 and Arg321 resulted in much less inhibition of SK2 by this inhibitor (Figure 2F). Alanine mutation at Phe154 had only a minor effect on SK2 inhibition by MP-A08 as it is shielded by the side-chain of Asn152 (Figure 2F), which was, again, in agreement with the docking. Surprisingly, mutation at Ser242 did not affect MP-A08 binding in SK2 despite the predicted hydrogen bonding interaction of its side-chain hydroxyl. It appears that for SK2 Arg315 and Arg321 tether MP-A08 in the pocket without the involvement of Ser242, whilst in SK1 Arg185, Arg191 and Ser112, all contribute to MP-A08 binding in this region of the ATP pocket.

### MP-A08 does not induce degradation of SK1

**Figure 3:**
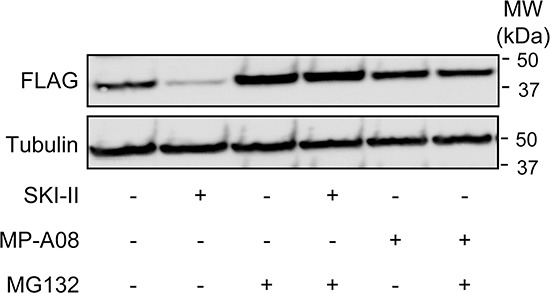
MP-A08 does not induce degradation of SK1 Western blots showing the effect of SKI-II (10 μM, 24 h) or MP-A08 (30 μM) treatment in the presence or absence of MG132 (10 μM, 24 h) on SK1-FLAG in HEK293 cells (upper panel). Tubulin was used as a loading control (lower panel). Results are representative of 2–3 independent experiments.

Some SK inhibitors, such as SKI-II and PF-543 have been found to inhibit SK1 in cells by targeting the protein for proteasomal degradation [33–36, 45]. Therefore we investigated whether MP-A08 induced proteasomal degradation of SK1. As reported previously [34], treatment with SKI-II caused a reduction in SK1 levels which could be restored with the proteasome inhibitor MG132 (Figure 3). Conversely, however, cell treatment with MP-A08 had no affect on SK1 protein levels, demonstrating that MP-A08 does not induce degradation of SK1 (Figure 3).

### MP-A08 inhibits S1P production in cells and increases pro-apoptotic sphingolipids

**Figure 4:**
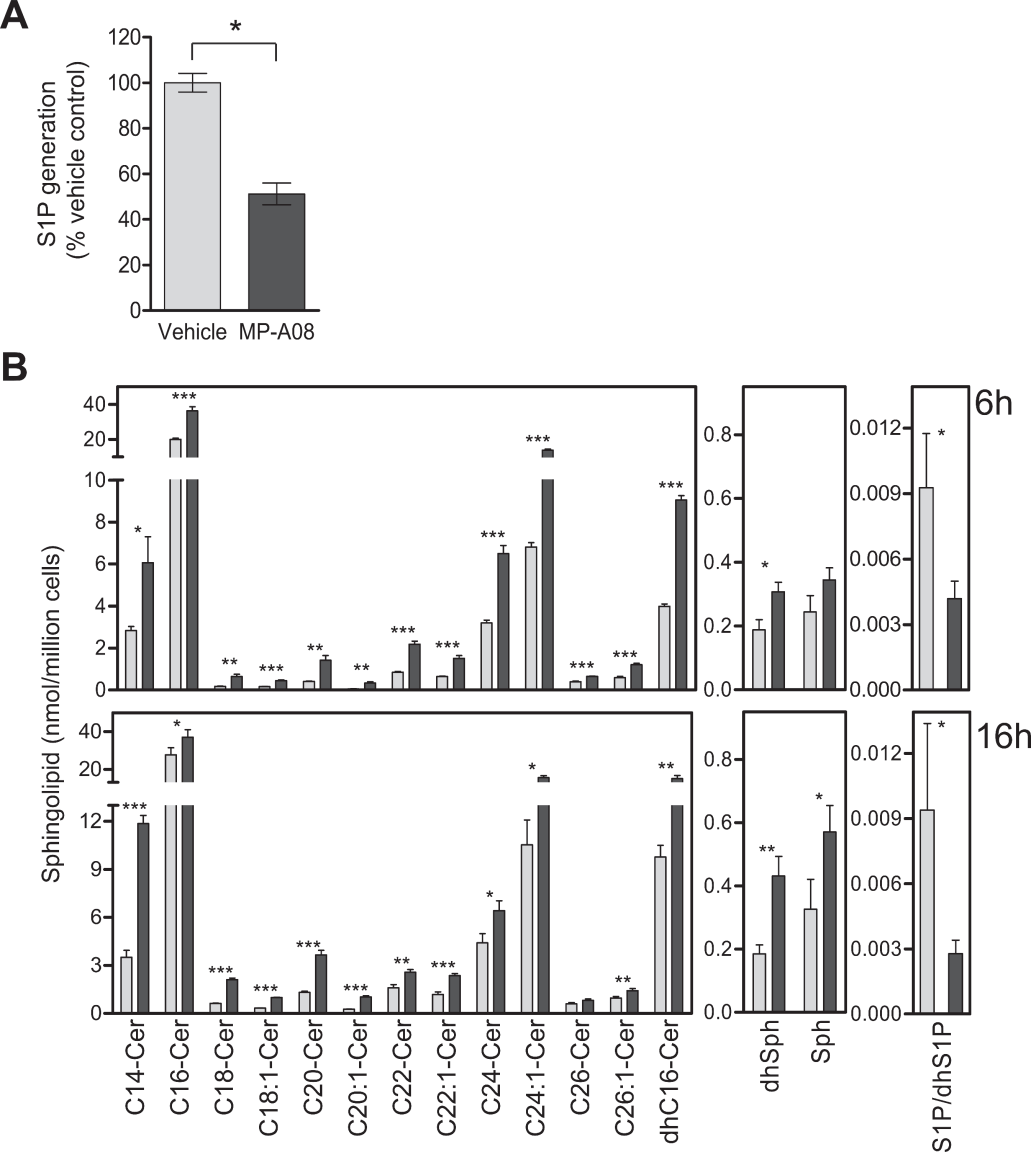
MP-A08 inhibits cellular S1P production, increases levels of sphingosine and ceramides, and alters cell signaling pathways associated with apoptosis and proliferation **(A)** The effect of MP-A08 on cellular S1P formation was determined in Jurkat cells as described in Materials and Methods, and presented as % vehicle control. Values are mean ± SD (*n* = 4). Significance was determined by student *t*-test (*p < 0.05). **(B)** Mass spectrometric analysis of sphingolipids from Jurkat cells treated with 15 μM MP-A08 (black bars) or vehicle control (grey bars) for 6 h (top) or 16 h (bottom). S1P levels in cell lysates were determined by SIP ELISA. Significance compared to vehicle control was determined by student *t*-test (*p < 0.05, **p < 0.01 and ***p < 0.001) (*n* = 3).

To test whether MP-A08 was a cell permeable SK inhibitor we next examined its ability to block S1P generation in cells. Treatment of Jurkat cells pre-labeled with ^3^H-sphingosine with 15 μM MP-A08 resulted in significantly reduced cellular S1P generation (Figure 4A), confirming this compound is cell permeable and able to block SK activity in cells. In order to assess the impact of MP-A08 on endogenous cellular sphingolipids the sphingolipid profile of MP-A08-treated Jurkat cells was analyzed (Figure 4B). Significant increases in levels of dihydrosphingosine, C16-dihydroceramide and all ceramides examined were observed at both 6 and 16 h after cell exposure to MP-A08 (Figure 4B). Indeed, cells treated with MP-A08 for 6 hours displayed significant increases in dihydrosphingosine and all ceramide species compared to vehicle-treated cells, but most notably in C18-, C20- and C20:1-ceramide, which displayed 3.7, 3.5 and 5.8-fold increases, respectively (Figure 4B). At 16 h after MP-A08 treatment the levels of C18-, C20- and C20:1-ceramides remained significantly elevated compared to vehicle controls (3.4, 2.8, 3.9-fold, respectively), while levels of C14- and C18:1-ceramide, dihydrosphingosine and sphingosine were elevated further compared to the 6 hour treatment. As expected, S1P levels were significantly decreased at both time points with 2-fold reduction at 6 h and a 3.5-fold reduction after 16 h.

### MP-A08 induces mitochondrial-mediated apoptosis

**Figure 5:**
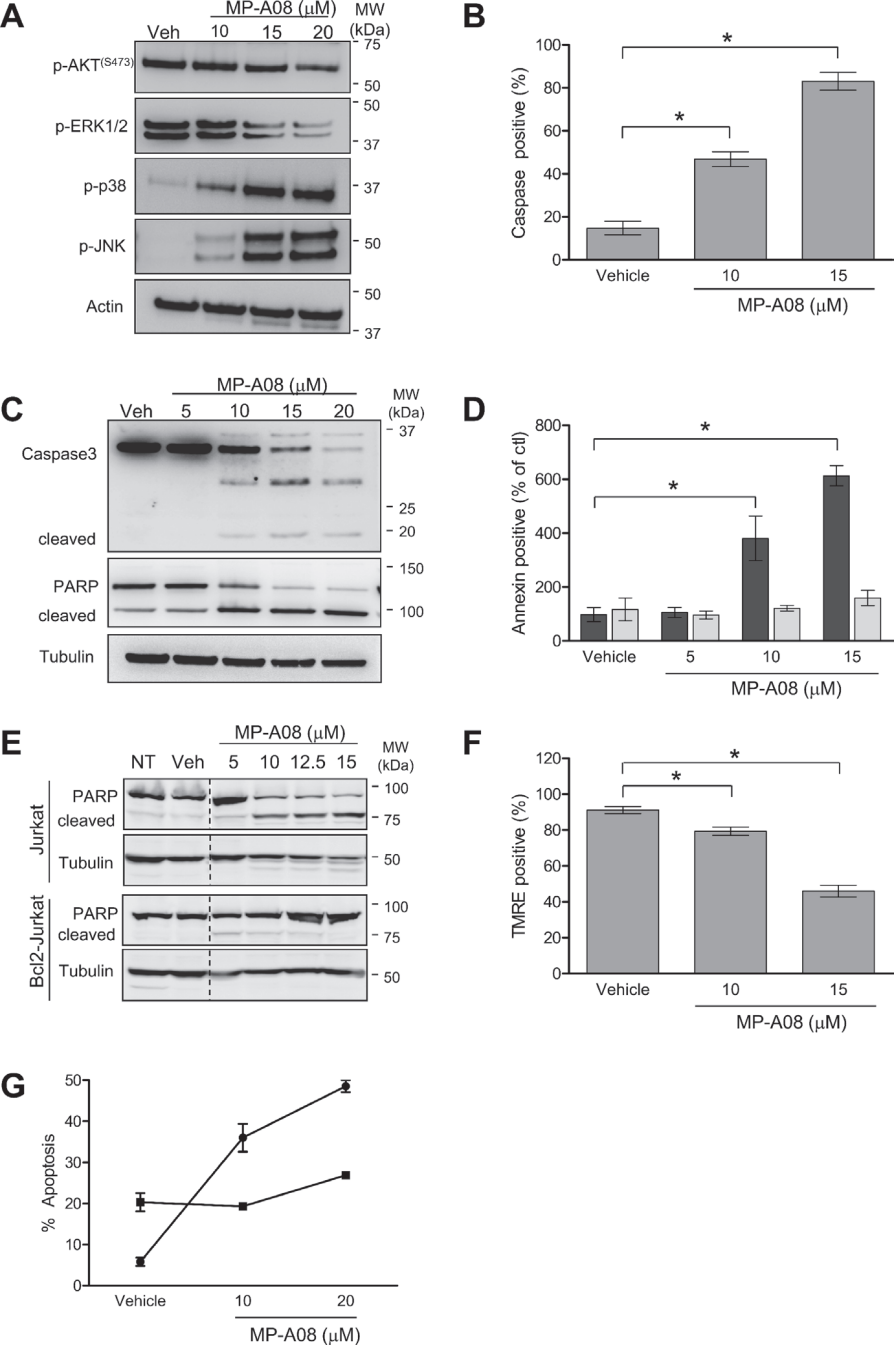
MP-A08 induces apoptosis in a sphingosine kinase-dependent manner **(A)** MP-A08 alters survival and proliferative signaling. Jurkat cells were treated with varying doses of MP-A08 (10–20 μM) for 6 h. Lysates were resolved by SDS-PAGE and analyzed by immunoblotting with antibodies against p-AKT(Ser473), p-ERK1/2, p-p38, and p-JNK (Cell Signaling). Actin was used as a loading control. Results are representative of two independent experiments. **(B)** Jurkat cells were treated with vehicle, 10 or 15 μM MP-A08 for 5 h and then assessed for caspase 3 activity (mean ± SD, *n* = 3; *p < 0.05 by Student's *t*-test). **(C)** Jurkat cells were treated with vehicle or MP-A08 (5–20 μM) for 6 h. Lysates were resolved by SDS-PAGE and analyzed for caspase 3 and PARP cleavage by immunoblotting with antibodies against caspase 3 and PARP, with tubulin used as a loading control. Results are representative of two independent experiments. **(D)** Jurkat (black bars) or Bcl2-expressing Jurkat cells (grey bars) were treated with MP-A08 (5–15 μM) or vehicle control for 24 h, and then stained with Annexin V to assess apoptosis. Values are represented as % of no treatment control (mean ± SD, *n* = 3; *p < 0.05 by Student's *t*-test). **(E)** Lysates from Jurkat or Bcl2-expressing Jurkat (Bcl2-Jurkat) cells treated with MP-A08 (5–15 μM) or vehicle control for 16 h were assessed for PARP cleavage by immunoblotting with PARP antibodies, with tubulin used as the loading control. Results are representative of two independent experiments. **(F)** Jurkat cells were treated with 10 or 15 μM MP-A08 or vehicle control for 5 h and stained with TMRE to test for changes in mitochondrial permeability (mean ± SD, *n* = 3; *p < 0.05 by Student's *t*-test). **(G)** Mouse embryonic fibroblasts from wildtype (●) or SK1/SK2 double knockout mice (■) were treated with MP-A08 or vehicle control overnight. Cells were stained with DAPI and assessed for apoptosis by visualizing nuclear condensation using confocal microscopy. Values are mean ± SD (*n* = 3).

As MP-A08 treatment was found to increase levels of pro-apoptotic ceramides we next assessed the affects of MP-A08 on signaling pathways associated with survival and proliferation. S1P is known to regulate the Akt and MAPK pathways via signaling through the S1P G-protein coupled receptors and via other unknown intracellular pathways [37]. As expected, MP-A08 treatment caused a dose-dependent loss in activation of the pro-survival and pro-proliferative Akt and ERK1/2 pathways, and induction of the apoptosis-associated p38 and JNK pathways (Figure 5A).

Previous studies have demonstrated that knockdown or inhibition of SKs induces apoptosis in many cell types [11, 38, 39]. Thus, we next assessed the effects of MP-A08 on induction of apoptosis via an array of apoptosis markers; caspase-3 cleavage and activation, PARP cleavage, Annexin V staining, and mitochondrial permeability transition by TMRE staining (Figure 5B–5F). Treatment of Jurkat cells with 10 and 15 μM MP-A08 caused a dose-dependent induction of caspase 3 activity (Figure 5B). This was confirmed by immunoblotting for caspase 3 and PARP cleavage products, which demonstrated a dose-dependent activation of caspase 3 and inactivation of PARP with MP-A08 treatment (Figure 5C). Consistent with this, MP-A08 also induced cell surface expression of annexin V in a dose-dependent manner (Figure 5D). To verify that MP-A08 was inducing mitochondrial-mediated apoptosis, Jurkat cells overexpressing the anti-apoptotic Bcl2 protein were treated with MP-A08 and assessed for apoptosis. Bcl2 overexpression blocked both MP-A08-induced annexin V staining and PARP cleavage compared to the parental cells (Figure 5D–5E). MP-A08 treatment of cells also reduced TMRE staining in a dose-dependent manner, indicating mitochondrial membrane permeabilization consistent with induction of mitochondrial-mediated apoptosis (Figure 5F).

To validate the SKs as the targets of MP-A08-induced apoptosis we next assessed the sensitivity of primary SK1/SK2 double knockout (dKO) mouse embryonic fibroblasts (MEFs) to MP-A08 treatment (Figure 5G). Unlike wildtype MEFs, which showed a dose-dependent increase in apoptosis with MP-A08 treatment, dKO MEFs had an elevated basal level of apoptosis that was unaffected by MP-A08 treatment (Figure 5G). These results strongly suggest that MP-A08 induced-apoptosis is mediated by inhibition of the SKs.

### MP-A08 blocks survival and neoplastic growth of a range of cancer cell lines

**Figure 6:**
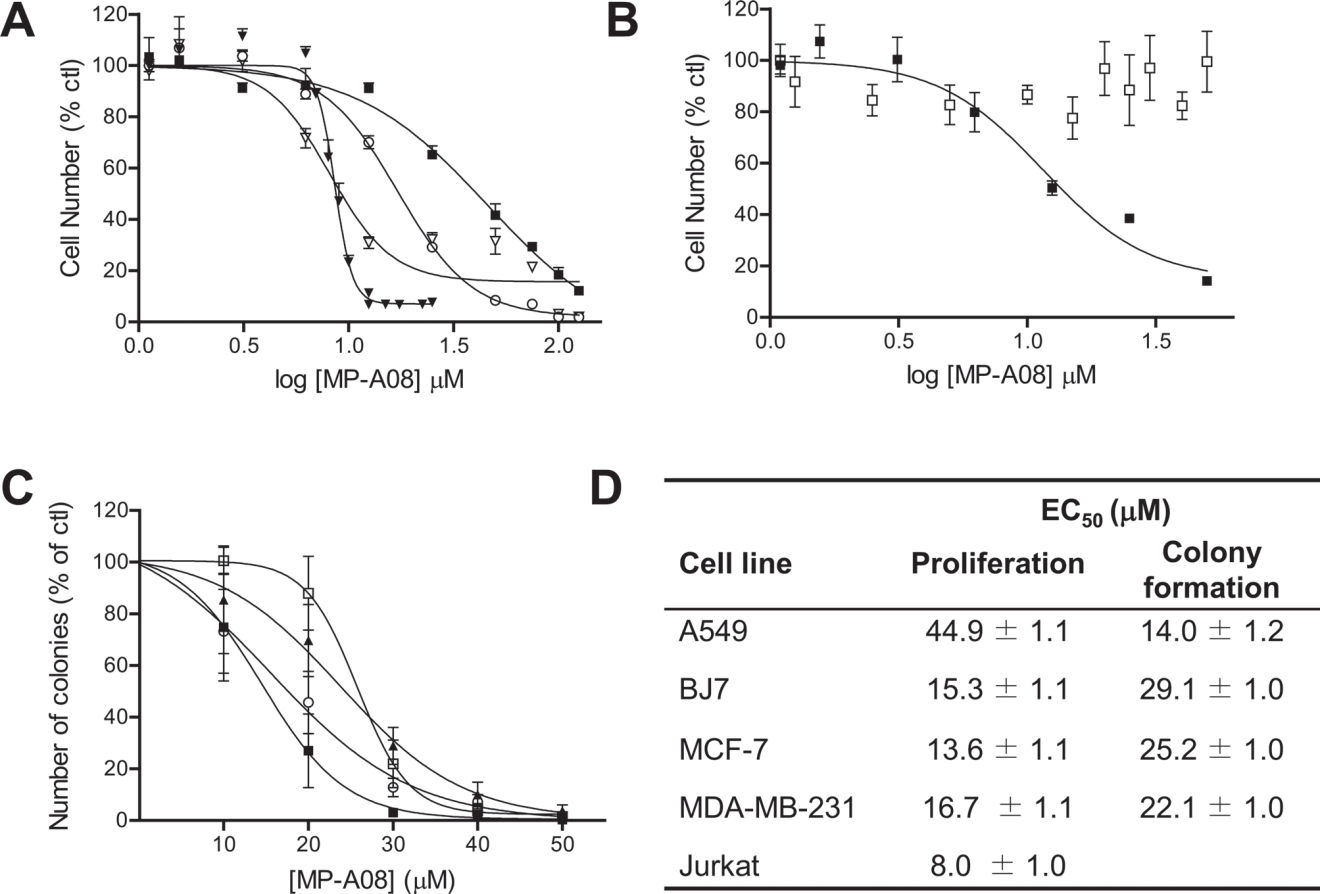
MP-A08 inhibits neoplastic growth of human cancer cells *in vitro* **(A)** For growth assays, values were determined for adherent cells lines by MTS assay and by flow cytometry for Jurkat cells (suspension) with varying concentrations (1.56–125 μM) of MP-A08 (48 h treatment). A549 (■), MCF7 (□), MDA-MB-231 (○), Jurkat (▼). Values are displayed as % vehicle control, mean ± SD (*n* = 3–4). **(B)** Growth of parental BJ1 (□) and transformed BJ7 (■) fibroblasts were determined by MTS assay with varying concentrations (1.25–50 μM) of MP-A08 (48 h treatment). Values are displayed as % vehicle control, mean ± SD (*n* = 3–4). **(C)** For colony formation assays, adherent cells lines A549 (■), BJ7 (▲), MCF7 (□), and MDA-MB-231 (○) were plated in 0.33% (final) low-melting point agarose with MP-A08 at varying concentrations (10–50 μM). This was overlaid onto 0.5% DMEM-low-melting agarose gel. Colonies were assessed by light microscopy and quantified using ImageJ after 14–21 days. Values are displayed as % vehicle control, mean ± SD (*n* = 3–4). **(D)** Curve-fitting for EC_50_ values for A–D were determined using non-linear regression analysis GraphPad (Prism) (*n* = 3–4, R^2^ > 0.95).

Due to the ability of MP-A08 to induce apoptosis in Jurkat cells we then tested its affects on the growth of a range of solid cancer cell lines, including three from the NCI60 human tumor cell line panel (A549 lung adenocarcinoma cells, and MCF-7 and MDA-MB-231 breast adenocarcinoma cells) (Figure 6A), as well as BJ7 human foreskin fibroblasts transformed via the expression of V12-Ras, the telomerase catalytic subunit and the SV40 large and small T antigens [40] and the parental untransformed foreskin fibroblast line, BJ1 (Figure 6B). These cell lines, along with Jurkat cells, were treated with varying doses of MP-A08 to determine the median effective concentration (EC_50_) to block cell proliferation (Figure 6A–6B, 6D). Growth of all five of the transformed cell lines (A549, MCF-7, MDA-MB-231, Jurkat and BJ7) was blocked by MP-A08, but with varying sensitivities (Figure 6A–6B, 6D), while notably the growth of the untransformed BJ1 fibroblast line was not affected by MP-A08 (Figure 6B). We next assessed the EC_50_ of MP-A08 required to block neoplastic, anchorage-independent growth of the solid tumor cell lines in *in vitro* colony formation assays, as a better indicator of anti-neoplastic activity. Again, MP-A08 blocked neoplastic growth of all four cell lines (Figure 6C–6D). Interestingly, however, the cell lines displayed very different sensitivities in the colony formation and cell growth assays. A549 cells, for example, were least sensitive to MP-A08 in cell proliferation assays, but most sensitive in colony formation assays (Figure 6C–6D).

### MP-A08 suppresses the growth of human lung tumor xenografts in mice

Prior to examining the anti-neoplastic effects of MP-A08 *in vivo*, we first sought to establish the maximal tolerated dose of MP-A08 in NOD/SCID mice. No adverse side effects were observed following daily i.p. administration of 50, 75 or 100 mg/kg MP-A08 to mice for 14 days through measurement of body weight, white cells, blood hemoglobin, or platelet numbers (Supplementary Figure 4). Furthermore, no differences were detected in tissue pathology of the kidney, liver, heart and spleen between these MP-A08 treated mice and vehicle treated mice, suggesting low toxicity of MP-A08.

**Figure 7:**
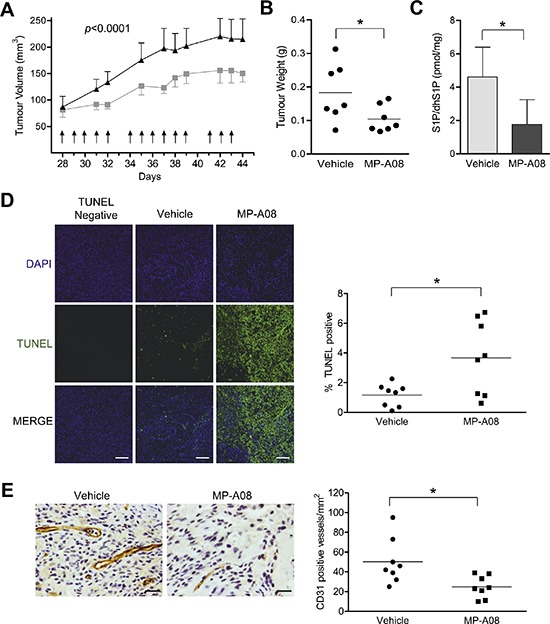
MP-A08 reduces tumor burden in an A549 xenograft mouse model **(A)** Mice bearing A549 xenografts were treated i.p. with 100 mg/kg MP-A08 or vehicle control at the times indicated (arrows). Mean tumor volumes ± SEM are shown (*n* = 8 per group). Statistical analysis on all data points was performed by two-way ANOVA. **(B)** After two weeks of treatment with MP-A08 (100 mg/kg) or vehicle control, tumors were excised and weighed (*n* = 8 per group, *p < 0.05 by Student's *t*-test). **(C)** S1P/dhS1P levels in the tumor homogenates were determined by S1P ELISA. Values are represented as mean +SD (*n* = 4–5), *p < 0.05 by Student's *t*-test) **(D)** MP-A08 induces apoptosis in xenograft tumors. Representative TUNEL-stained xenograft sections from MP-A08 or PEG treated mice as presented in **(A–B)**. A representative 40× image is shown. The scale bar indicates 100 μM. Image intensity was quantified using ImageJ (right) (*n* = 8 per group, *p < 0.05 by Student's *t*-test). **(E)** MP-A08 reduces tumor angiogenesis. Representative CD31-stained tumor sections from MP-A08 or vehicle control-treated mice as presented in **(A–B)**. CD31 positive vessels per field of view were quantitated (*n* = 8 per group; *p < 0.05 by Student's *t*-test). The scale bar indicates 50 μM.

We next examined *in vivo* efficacy of MP-A08 to attenuate growth of A549 human lung adenocarcinoma xenografts in mice. Subcutaneous xenografts of A549 cells were established in NOD/SCID mice and grown to a volume of approximately 75 mm^3^ before mice were treated with MP-A08 at 100 mg/kg, six times a week for two weeks. MP-A08 significantly reduced tumor volume and weight (Figure 7A–7B). Consistent with *in vivo* SK inhibition, excised tumors from MP-A08 treated mice displayed significantly lower S1P compared to the tumors from vehicle control mice (Figure 7C). MP-A08 treated tumors exhibited significantly higher levels of apoptotic cell death compared to the vehicle control as indicated by TUNEL staining (Figure 7D). In addition to promoting cell growth and survival, SK has an established role in promoting cancer angiogenesis [41]. As expected, MP- A08 treated tumors showed reduced vasculature as signified by a reduction in CD31 positive blood vessels (Figure 7E). This data, therefore, suggests MP-A08 acts as an anti-cancer agent by both inducing apoptosis and blocking angiogenesis.

## DISCUSSION

In this study we have identified MP-A08 as a novel, first-in-class selective ATP-competitive inhibitor of SK1 and SK2. MP-A08 alters the balance of the sphingolipid rheostat away from anti-apoptotic, pro-proliferative S1P, towards pro-apoptotic sphingosine and ceramide, and as a result blocks neoplastic growth and induces apoptosis in a range of human cancer cell lines. Furthermore, MP-A08 is active *in vivo*, inhibiting the growth of human lung adenocarcinoma xenograft tumors in mice via a mechanism that involved both induction of tumor cell apoptosis and reduction in tumor angiogenesis.

Previously developed SK inhibitors have almost exclusively targeted the sphingosine-binding pocket of these enzymes. As these inhibitors often retain sphingosine-like characteristics, just like sphingosine, they can have off-target affects [11, 20, 22–24]. The sphingosine-analog inhibitor SKI-II, for example, inhibits dihydroceramide desaturase, an enzyme in the *de novo* sphingolipid synthesis pathway [22]. Similarly, the sphingosine-like FTY720, which shares a common backbone with other sphingosine-analog inhibitors, can at high concentrations disrupt membranes, inhibit other sphingosine-binding or lipid metabolizing enzymes such as ceramide synthase and S1P lyase, and also target protein phosphatase 2A, the pro-survival 14–3-3 protein, and autotaxin [42]. For this reason we sought ways to target the ATP pocket of SK1 for inhibitor development; a strategy employed with success with many protein kinase inhibitors despite these proteins possessing a very high degree of structural conservation in their ATP binding sites [43, 44]. For the SKs, this approach was particularly attractive as their ATP-binding pockets are structurally distinct from protein kinases, as well as almost all other lipid kinases [28, 32], allowing it to be effectively exploited for inhibitor development.

Until very recently no structural information was available for the SKs. Therefore, we employed a homology modeling and *in silico* docking approach to screen for inhibitors targeting the ATP-binding pocket of SK1. Notably, retrospective comparison showed our predicted SK1 model aligned very closely with the recently published SK1 crystal structure [32]. Using modeling and mutagenesis we have built on the mechanistic understanding of catalysis by the SKs obtained from the SK1 crystal structure by characterizing the residues required for ATP binding and catalysis of SK1. Of note, our analysis demonstrated the requirement of residues from all five regions highly conserved in the SKs; ^21^LNPRGG^26^, ^54^TERR^57^, ^79^SGDGLMHE^86^, ^111^GSGN^114^ and ^340^VDGE^343^ (where underlined residues denote where mutation reduced or abolished SK1 activity). Furthermore, our prediction and mutagenesis of the ATP-binding pocket of SK2 confirmed its high overall conservation with that of SK1, but also uncovered key differences in this region between the two human SK isoforms that may allow for the development of isoform-selective ATP-competitive inhibitors of these enzymes.

Identification and subsequent analysis of MP-A08 inhibition revealed it to act, as expected, as an ATP directed inhibitor of SK1. MP-A08 also inhibited SK2 in a comparable manner, although surprisingly with a somewhat higher affinity than SK1. Analysis of MP-A08 docking into the SK1 structure and SK2 model suggests that the Phe154 and Asn187 substitutions in the ATP-binding pocket of SK2 (compared to Arg24 and Arg57 in SK1, respectively) may explain, in part, the increased MP- A08 affinity for SK2. These amino acid substitutions in the SK2 pocket are predicted to cause an altered arrangement of the phenyl rings of MP-A08, binding the molecule deeper into the pocket. Interestingly, inhibition of SK1 by MP-A08 did not induce proteasomal degradation. This is in contrast to the sphingosine-analog inhibitors SKI-II [34], PF-543 and 55–21 [45]. Therefore it is tempting to speculate that inhibitor-induced degradation is a property regulated by binding to the sphingosine pocket and not the ATP pocket of SK1.

Consistent with the divergent structure of the ATP-binding pocket of SK1 from protein kinases, MP-A08 displayed a high degree of specificity for the SKs. This is in stark contrast to CB5468139, the only other ATP-binding site directed SK1 inhibitor described to date, which inhibited a broad array of protein kinases [46]. Indeed, TSSK1 appeared the only potential off-target of MP-A08 in our kinome screen, although the *in vitro* inhibition of this protein kinase by MP-A08 was only modest, even when the inhibitor was employed at 250 μM. Notably, TSSK1 expression is restricted to the testis, and thus any potential inhibitory effect on this protein kinase is unlikely to impact on the findings of this study. Furthermore, due to its role in spermatid maturation, TSSK1 has been targeted for contraceptive development [47], meaning any off-target inhibition of this enzyme is unlikely to present serious detrimental side-effects *in vivo*. Interestingly, our studies also showed that Ser79, Leu83 and Ser112 of SK1 contribute to MP-A08 binding. Since these residues are not conserved in CERK and DAGK, this is consistent with these residues conferring selectivity of MP-A08 for the SKs over other related members of the SK/DAGK superfamily.

It is notable that MP-A08 significantly increased the cellular levels of sphingosine, dihydrosphingosine and all ceramide and dihydroceramide species examined (Figure 4B). Unlike SKI-II which displays off-target inhibition of dihydroceramide desaturase, increases in dihydroceramides with MP-A08 treatment are likely induced by ceramide accumulation and re-equilibration of all upstream pre-cursors (including dihydroceramides and dihydrosphingosine). Our findings with MP-A08 are consistent with almost all previous studies that have found significant increases in ceramide levels following genetic ablation, or transient knockdown or inhibition of SK1, with this correlating with induction of apoptosis [38, 39, 48, 49]. This is, however, in stark contrast to some recently reported high affinity SK inhibitors that fail to induce apoptosis or show anti-neoplastic effects [25–27]. Notably, where examined, these high affinity inhibitors did not show any enhanced ceramide levels when used at concentrations sufficient to inhibit SK1 [25, 27]. This, combined with observed increases in sphingosine levels by these inhibitors, which would normally be associated with enhanced ceramides [21, 38, 39, 46, 50, 51], suggest these high affinity inhibitors may exhibit inhibitory effects on at least some ceramide synthases [45], potentially explaining their impotence as anti-neoplastic agents.

Examination of the downstream signaling affects of SK1/2 inhibition by MP-A08 revealed a dose-dependent reduction in phospho-Akt and phospho-ERK1/2 (Figure 5A), indicating a dampening of the phosphoinositide 3-kinase/Akt and Ras/ERK1/2 pathways, respectively. MP-A08 concomitantly induced a dose-dependent activation of stress pathways through phospho-p38 and phospho-JNK (Figure 5A). This loss of pro-survival and pro-proliferative signaling and induction of stress-induced signaling, combined with induction of numerous markers of apoptosis, is completely in agreement with known effects of blockade of the SK pathway [21, 38, 39, 46, 50, 51]. Furthermore, the absence of effects of MP-A08 on MEFs lacking SK1 and SK2 provides considerable evidence that the observed cellular effects of this molecule are mediated via SK1/2 inhibition. Interestingly, transient SK inhibition in wildtype MEFs resulted in greater apoptosis than that observed with genetic ablation of the SKs in the dKO MEFs. While the reasons for this remains unclear, it is possible that the chronic loss of SKs in the dKO cells results in selection of a population of MEFs that is somewhat more resistant to apoptosis.

Examination of the sensitivities to MP-A08 of different cancer lines showed both reduced cell proliferation and *in vitro* neoplastic growth, with EC_50_ values comparable to those of a number of other widely employed SK inhibitors [46, 52]. In addition, MP-A08 did not affect the growth of the parental untransformed BJ1 fibroblast cell line, while the transformed BJ7 cells displayed a dose-dependent reduction in cell growth with treatment (Figure 6B). This suggests that transformed cells are more sensitive to MP-A08 consistent with previous non-oncogene addiction theories for the SKs [53, 54].

With A549 human lung adenocarcinoma cells demonstrating sensitivity to MP-A08 we undertook a mouse xenograft study with these cells, with MP-A08 significantly reducing tumor growth, demonstrating clear efficacy of this inhibitor *in vivo*. Indeed, MP-A08 enhanced tumor apoptosis, and consistent with the known role of SK1-derived S1P as a potent angiogenic factor through S1P_1_ receptor [55–57], also reduced tumor vascularization (Figure 7D).

In conclusion, here we report a first-in-class ATP-binding site-directed small molecule SK inhibitor, MP-A08 that was discovered using a combined approach of structural homology modeling of the ATP-binding site of SK1 and *in silico* docking with small molecule libraries. MP-A08 is a highly selective ATP-competitive inhibitor that inhibits both SK1 and SK2. MP-A08 blocks pro-proliferative signaling, induces apoptosis in an SK-dependent manner and represses the growth of human lung adenocarcinoma in a mouse xenograft model by both inducing tumor apoptosis and inhibiting angiogenesis. Therefore, this newly developed SK inhibitor that targets the ATP-binding pocket provides a promising candidate for further development as a potential cancer therapy.

## MATERIALS AND METHODS

### Prediction of SK structures by homology modeling

A multiple sequence alignment of human DAGKα (Genbank Accession number: NP_963848), human DAGKζ (NP_963290), human CERK (NP_073603), *Escherichia coli* YEGs (P76407), *Staphylococcus aureus* DgkB (Sequence from PDB code: 2QV7) and human SK1a (Q9NYA1) was performed using ClustalW [58]. This alignment was used as input for Modeller 9v6 [59]. DgkB (2QV7) and YegS (2JGR) crystal structures were used as templates for homology modeling. Due to poor homology with the template structures, residues 1–12, 162–189 and 203–230 of human SK1 were excluded from the SK1 model. The model was optimized using 50 cycles of Refmac structure idealization [60]. The CCP4 software suite was used for model assessment and structural alignments using Procheck and Superpose modules, respectively [60]. Figures were produced using the Pymol graphics program [61]. The structural model of human SK2 was produced as described above using the human SK1 crystal structure [32] bound with ADP (3VZD) as a template. Due to large insertions in the SK2 sequence (AAH10671) residues 1–142, 355–370 and 609–618 were excluded from the SK2 model.

### *In silico* molecular screening

The SK1 structural model and DgkB crystal structure were prepared for docking using the DockPrep module in Chimera [62]. Docking was performed using DOCK6 [63, 64] on the eResearch SA Hydra server. Docking parameters were optimized using comparisons of the structure of DgkB co-crystallized with ADP and with the DgkB structure docked with ADP. For *in silico* screening, a virtual library of 120,000 compounds was generated from databases (Sigma-Aldrich, Calbiochem, and the National Cancer Institute Chemical and Natural Products libraries) [65]. The docking was carried out in two stages: initial low stringency screening, and then high stringency screening. Default high stringency docking parameters were used with a minimum anchor size of two atoms and scored with chemical matching. All compounds that docked at high stringency were assessed by score, rank and visual assessment. Candidates were chosen for biological testing and were sourced from the Drug Synthesis and Chemistry Branch, Developmental Therapeutics Program, Division of Cancer Treatment and Diagnosis, National Cancer Institute, USA.

### Chemical synthesis

MP-A08 was synthesized, purified and identity verified by ChemBridge Inc (San Diego, USA) with > 95% purity.

### Construction of SK mutants

FLAG-tagged human SK1 and SK2 constructs in pcDNA3 vector (Invitrogen) have been previously described [66, 67]. Quikchange^®^ PCR mutagenesis was carried out with forward and reverse mutagenic oligonucleotides (Supplementary Table 2). DNA sequencing verified the integrity of all mutated cDNAs.

### Cell culture

HEK293 (human embryonic kidney, 293c18 cells, ATCC# CRL-10852), A549 (human lung adenocarcinoma, ECACC# 86012804), untransformed parental (BJ1) and transformed human foreskin fibroblasts (BJ7) [40], MCF7 (human mammary adenocarcinoma, ECACC# 86012803) and MDA-MB-231 (human mammary adenocarcinoma, ATCC# HTB-26) cells were cultured in Dulbecco's modified Eagle's medium (Gibco, Invitrogen), containing 10% fetal bovine serum (Bovagen), 2 mM glutamine, 0.2% (w/v) sodium bicarbonate, 1 mM HEPES, penicillin (1.2 mg/ml) and streptomycin (1.6 mg/ml). Jurkat (human T cell lymphoblast) and Jurkat-Bcl2 cells were cultured in suspension in RPMI medium containing 10% fetal bovine serum (Bovagen), 2 mM glutamine, 0.2% (w/v) sodium bicarbonate, 1 mM HEPES, penicillin (1.2 mg/ml) and streptomycin (1.6 mg/ml). All cells were grown at 37°C, 5% CO_2_ in a humidified incubator.

SK1/SK2 double knockout MEFs were generated from timed matings of female SK1^+/−^/SK2^−/−^ mice with male SK1^−/−^/SK2^+/−^ mice. Fibroblasts from 11.5 day post coitum embryos were isolated and cultured in DMEM containing 10% bovine calf serum (Bovogen), penicillin (1.2 mg/ml) and streptomycin (1.6 mg/ml) at 37ºC in a humidified atmosphere with 10% CO_2_. Cells were genotyped to identify cultures with SK1/SK2 double knockout genotype. Wild-type MEFs were generated from 14.5 day post coitum embryos and cultured as described above.

### Assaying SK ATP-binding pocket mutants for activity

HEK293 cells were seeded in 6-well plates and were transiently transfected using Lipofectamine^TM^ 2000 Transfection Reagent (Invitrogen) according to the manufacturer's protocol. Cells were harvested 24 h post-transfection, and cell pellets were resuspended in 50 mM Tris-HCl buffer (pH 7.4) containing 150 mM NaCl, 10% glycerol, 1 mM EDTA, 0.05% Triton X-100 (excluded for SK2 samples), 2 mM Na_3_VO_4_, 10 mM NaF, 10 mM β-glycerophosphate, 1 mM dithiothreitol and protease inhibitor cocktail (Roche). Cells were lysed by sonication and diluted in extraction buffer for assays (1:1000 final dilution). Expression levels of FLAG-tagged SK1/2 proteins were assessed by SDS-PAGE and anti-FLAG immunoblotting. SK1 and SK2 activity was determined using d-*erythro*-sphingosine and [γ^32^P] ATP as substrates, as described previously [68].

### Generation of recombinant proteins in insect cells

Baculovirus SK1 expression constructs encoding for human SK1a with a c-terminal TEV-cleavable 6xHis tag was generated by PCR with oligonucleotide primers

5′-TAGAATTCGCCACCATGGATCCAGCGGGC GGC-3′ and

5′-TAGAATTCTCAGTGATGGTGATGGTGATGT TCCAGGCCCTGAAAATACA

GGTTTTCTAAGGGCTCTTCTGGCGGT-3′ using pcDNA3-SK1 [66] as a template. The resultant product was cloned into pFastBac1 (Invitrogen) by digestion with EcoRI. Recombinant human SK1 protein was expressed using the baculovirus expression system in Sf9 cells and purified as previously described [28].

Purified recombinant human SK2 protein with 6xHis and 3xhemagglutinin (HA) tags was generated in Sf9 cells and purified as detailed previously [67].

Human ceramide kinase cDNA (Genbank Accession number NM_022766) was amplified from human placenta cDNA with primers: 5′-TAGGATCCG CCACCATGGGGGCGACGGGGGC-3′ and 5′-TAGAA TTCTCAGTGATGGTGATGGTGATGTTCCAGGCCCT GAAAATACA

GGTTTTCGCTGTGTGAGTCTGGCTTC-3′, and cloned into pFastBac1 following digestion with BamHI and EcoRI. Human DAGK cDNA (DAGKα; NM_201444) was PCR amplified from human foreskin fibroblast cDNA with primers

5′-TAGGATCCAAGCTTGCCACCATGGCCAAG GAGAGGGGCC-3′ and

5′TAGGTACCAAGCTTCAGTGATGGTGATGG TGATGTTCCAGGCCCTGAAA

ATACAGGTTTTCGCTCAAGAAGCCAAAGAA ATTG-3′. The PCR product was digested with HindIII and cloned into pFastBac1. Sequencing verified the orientation and integrity of all the cloned cDNAs. Recombinant bacmids and baculoviruses were produced according to the manufacturers’ protocols. To generate CERK and DAGKα proteins Sf9 cells (2 × 10^9^ cells) were infected with the recombinant baculovirus (MOI 5–10) for 96 h. Infected cells were harvested and snap frozen and stored at –80°C until required. Cell pellets were resuspended in Buffer A (50 mM Tris-HCl buffer pH 7.6, 150 mM NaCl, 10% glycerol (w/v), 40 mM imidazole, and protease inhibitors (EDTA-free Complete™, Roche). Triton X-100 was added to a final concentration of 1% (v/v) and the cell suspension incubated on ice for 30 min. The lysate was clarified by centrifugation at 50,000 × g for 20 minutes at 4°C. The resulting lysate was incubated with 1.5 ml Buffer A-equilibrated Nickel-NTA sepharose (GE Healthcare) and incubated at 4°C for 30 minutes with shaking. The Nickel-NTA was packed into a column and washed with 5 column volumes of Buffer A. The protein was eluted with Buffer A containing 0.2 M imidazole.

### MP-A08 cross-screening

SK1 and SK2 activity was determined using d-*erythro*-sphingosine (solubilized in fatty acid-free bovine serum albumin) and [γ^32^P] ATP as substrates, as described previously [68]. DAGK assays were performed using β-octylglucoside solubilized dioleoyl-s,n-glycerol (DAG) and phosphatidylserine (PS), based on methods previously described [69]. CERK assays were performed as described previously [70]. Cross-screening assays were carried out using 20 μM ATP (0.5 μCi [γ^32^P]ATP) and contained either MP-A08 (0.1 mg/ml, 192 μM) or vehicle control (1% DMSO/9% (v/v) ethanol final). Data are represented as % activity compared to vehicle control. For determination of kinetic constants SK assays were carried out as described above with 7.8–500 μM ATP and 100 μM sphingosine and treated with either vehicle (0.25% DMSO/2.25% (v/v) ethanol), 25 or 50 μM MP-A08. Kinetic constants were calculated using non-linear regression in Graphpad Prism 5.

Screening of MP-A08 for inhibitory activity against a panel of 140 protein kinases encompassing the major enzymes within this protein family was performed at the Dundee International Centre for Kinase Profiling. Dihydroceramide desaturase assays were carried out in intact Jurkat cells as described previously [71].

### SK1 degradation assays

Flp-In T-Rex HEK293 cells (Invitrogen) with doxycycline-inducible expression of FLAG-tagged human SK1 were generated as previously described [34, 72]. Expression of wild-type SK1 in Flp-In T-Rex HEK293 cells was induced with low concentrations of doxycycline hyclate (50–200 ng/ml) that resulted in approximately 10-fold increases in SK1 activity above basal levels. After 24 h, cells were treated with 10 μM SKI-II or 30 μM MP-A08 with or without 10 μM MG132. PEG400 0.35% (v/v), 0.58% (v/v) DMSO was used as the vehicle control. Cells were harvested after 24 h treatment and assessed for SK1 levels by SDS-PAGE and immunoblotting using anti-FLAG (Sigma) or anti-Tubulin (Abcam) antibodies, as described previously [34].

### Sphingolipid analysis

S1P generation in cells was assessed in Jurkat cells suspended at 5 × 10^5^ cells in 1 ml RPMI containing 0.5% FBS. Cells were treated with 15 μM MP-A08 or vehicle control (0.06% (v/v) DMSO/0.54% (v/v) ethanol) for 4.5 h at 37°C and 5% CO_2_. Cells were labeled with 0.5 μCi ^3^H-sphingosine (Perkin Elmer), incubated for 30 min at 37°C and 5% CO_2_, and then harvested by centrifugation at 1500 × g for 1 min, washed once with cold phosphate-buffered saline. Cells were lysed in 300 μl of acidified methanol (methanol:HCl, 100:1, by vol.), and S1P (including ^3^H-S1P) extracted by the addition of 300 μl of chloroform, 300 μl of 2M KCl and 30 μl of 3M NaOH. The samples were mixed and then centrifuged at 13000 × g for 5 min to separate the chloroform and aqueous/methanol phases, which under these alkaline conditions contained the partitioned ^3^H-sphingosine and ^3^H-S1P, respectively. ^3^H-S1P in the upper aqueous/methanol phase was then determined by scintillation counting.

Sphingolipid mass spectrometric analyses analysis was performed on 5 × 10^6^ Jurkat cells treated in the same manner as above for 6 h or 16 h with either 15 μM MP-A08 or vehicle control. For mass spectrometric analyses cells were pelleted, lyophilized and sphingosine, ceramides and their dihydro species assessed at the Medical University of South Carolina Lipidomics Core Facility using methods described previously [73]. S1P/dhS1P levels were assessed by ELISA (MyBioSource, MBS069092). Cell pellets were prepared according to the manufacturer's instructions.

### Assessment of apoptosis, cell viability, and proliferation

Jurkat cells (5 × 10^5^ cells/ml) were treated with vehicle (70% PEG, 0.28% v/v final) or MP-A08 (5–15 μM) for 5 or 24 h in RPMI medium containing 0.5% FBS. Analysis of TMRE staining and caspase 3 activity were carried out as described previously [74]. Annexin V staining was carried out according to manufacturer's instructions (Annexin-V-Fluos, Roche) and analyzed by flow cytometry as described previously [74]. Generation of the Jurkat cell line stably expressing human Bcl-2α will be described elsewhere.

For population cell growth assays, adherent cancer cell lines (A549, BJ1, BJ7, MCF7 and MDA-MB-231) were seeded into 48-well plates (20,000 cells per well) or 96-well plates (15,000 cells per well) 8 h prior to treatment. Cells were then treated with MP-A08 or vehicle control (0.16% (v/v) DMSO/1.44% (v/v) ethanol) in DMEM containing 0.5% FBS, 1.2 mg/ml penicillin, 1.6 mg/ml streptomycin, and 1 mM HEPES. After 48 h relative viable cell numbers were determined using the MTS assay (Promega) according to the manufacturer's protocol. Apoptosis in MEFs was assessed by DAPI staining as previously described [72].

### Colony formation in soft agar

Assays were performed as previously described [75], with the following modifications. Cells were prepared in growth media containing 0.33% DMEM-low-melting point agarose (Sigma) with either MP-A08 or vehicle control. This was overlaid onto 0.5% DMEM-low-melting agarose gel. After 14–21 days cells were analyzed by light microscopy and the number of colonies was quantified using ImageJ software [76].

### Toxicity study of MP-A08 in NOD/SCID mice

MP-A08, dissolved in 70% (v/v) polyethylene glycol 400 (PEG 400), was administered at 50, 75 and 100 mg/kg by intraperitoneal (i.p.) injection daily for 2 weeks, as was vehicle control. Mice were weighed daily, and murine cell blood counts were determined after two weeks of treatment by a SYSMEX XE 2100 hematology analyzer. Tissue pathology of hematoxylin and eosin stained sections of the kidney, liver, heart and spleen of MP-A08 and vehicle-treated mice were assessed by an experienced veterinary pathologist.

### A549 xenograft model

Mice were used with permission from the SA Pathology/Central Adelaide Local Health Network Animal Ethics Committee and experiments were performed under guidelines from the Australian code of practice for the care and use of animals for scientific purposes 7th Edition, 2004.

A549 cells (5 × 10^6^) were subcutaneously injected into the flanks of 6–8 week old female NOD/SCID mice. Tumor development was assessed daily and measured by caliper. Four weeks post engraftment when tumor sizes reached approximately 50–100 mm^3^, MP-A08 was administered at 100 mg/kg six days a week for 2 weeks, with daily measurement of tumors. Tumors were then excised and half was fixed in 10% formalin, paraffin embedded and sectioned. The remaining tumor was homogenized in a 1.5 ml tube using a pestle (Axygen) in 20 mM Tris-HCl (pH 7.4), 20% glycerol, 1 mM β-mercaptoethanol, 1 mM EDTA, 1 mM sodium orthovanadate, 15 mM NaF, 0.5 mM deoxypyridoxine, 40 mM β-glycerophosphate and protease inhibitor cocktail (Roche, EDTA-free) and tissue debris was pelleted by centrifugation at 13,000 × g for 15 mins at 4°C. S1P levels in samples were assessed by a S1P ELISA assay kit (Echelon; K-1900) according to manufacturers’ instructions. Apoptosis was examined by TUNEL staining using the Fluorescein *in situ* cell death detection kit (Roche) according the manufacturers’ instructions. For immunohistological analysis of visualization tumor tissue sections underwent a citrate buffer antigen retrieval process followed by blocking with 10% serum/PBS at room temperature for 60 min. Affinity purified goat polyclonal antibody to CD31/PECAM-1 (Santa Cruz; sc-1506) at 0.2 μg/ml was incubated overnight at 4°C followed by a 35 min incubation with biotinylated rabbit anti-goat antibody (1:500; Abcam) at room temperature. Sections were then incubated with VECTASTAIN Elite ABC Reagent at room temperature for 30 min, followed by peroxidase substrate solution. Sections were counterstained with Mayer's haematoxylin, mounted using DPX and visualized on an Olympus BX45 microscope equipped with an XC10 camera. A single image was collected using the 10× objective which covered 30–60% of the total area of the section, and CD31-immunoreactive vessels were enumerated by a person blinded to the identity of the samples.

## SUPPLEMENTARY FIGURES AND TABLES



## Abbreviations

CERK: ceramide kinase
DAGK: diacylglycerol kinase
DAPI: 49–6-Diamidino-2-phenylindole
dKO: double knock-out
HA: hemagglutinin
MEF: mouse embryonic fibroblasts
RMSD: root mean square deviation
S1P: sphingosine 1-phosphate
SK: sphingosine kinase
TMRE: tetramethylrhodamine ethyl ester
TSSK: testis-specific serine kinase
