# Supplementary material for: A selective ATP-competitive sphingosine kinase inhibitor demonstrates anti-cancer properties
Source: Oncotarget. 2015 Mar 11;6(9):7065–83. doi: 10.18632/oncotarget.3178 (PMC4466670; doi:10.18632/oncotarget.3178)
Supplement: Supplementary file 1 [file oncotarget-06-7065-s001.pdf]

SUPPLEMENTARY FIGURES AND TABLES

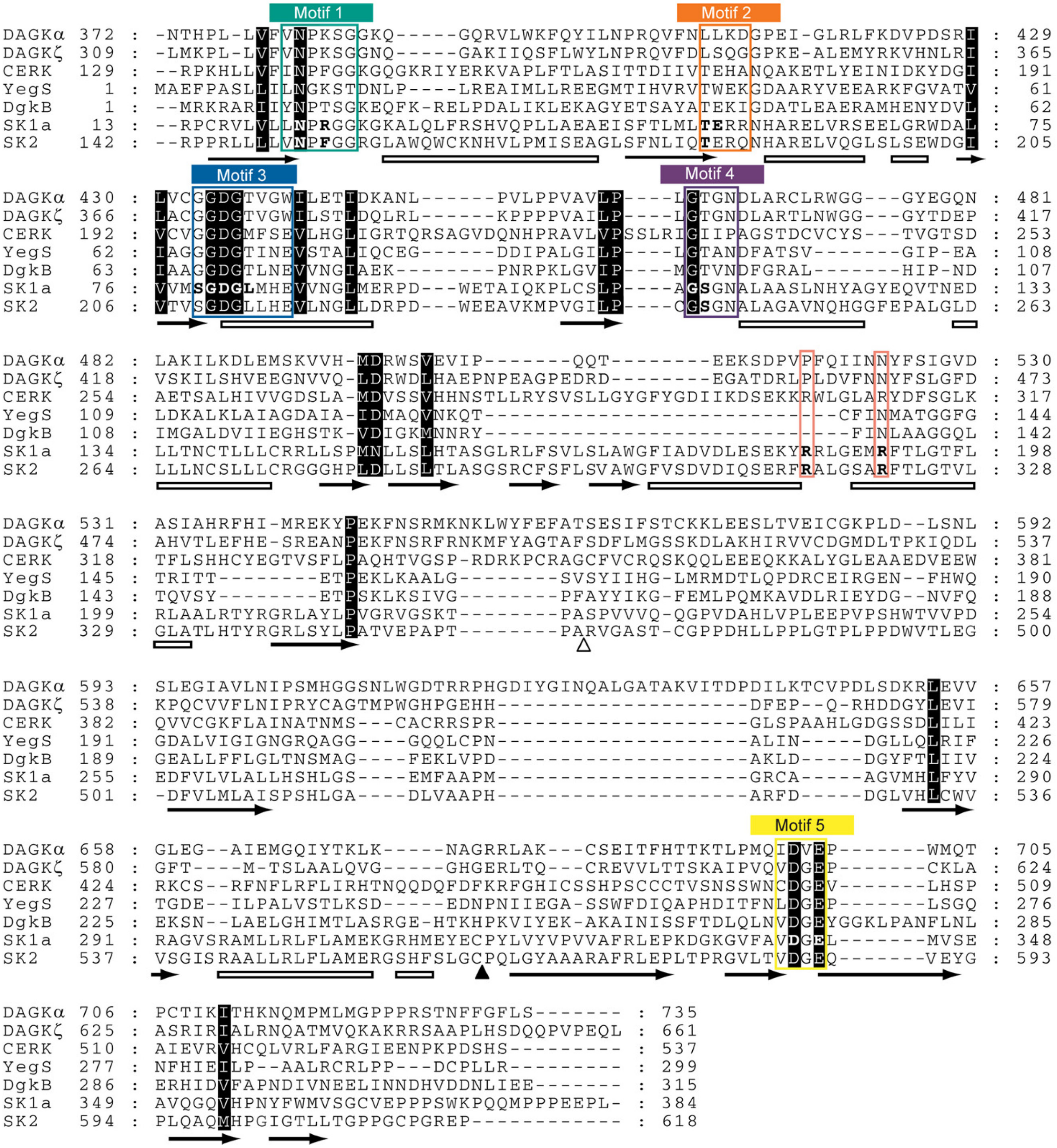

Supplementary Figure 1: Multiple sequence alignment used as input for SK homology modelling. A multiple sequence alignment of human DAGKα (Genbank Accession number: NP_963848), human DAGKζ (NP_963290), human CERK (NP_073603), *Escherichia coli* YEGs (P76407), *Staphylococcus aureus* DgkB (Sequence from PDB code: 2QV7), human SK1a (Q9NYA1) and SK2 (AAH10671) was performed using ClustalW [58]. This alignment was used as input for Modeller 9v6 [59]. DgkB (2QV7) and YegS (2JGR) crystal structures were used as templates for homology modelling. A large loop section (residues 447–477, denoted by ▲) was removed from CERK and the first 142 and a large internal loop section (residues 355–370, denoted by Δ) was removed from the SK2 sequence to aid the alignment. SK1 and SK2 secondary structure is represented with symbols: open rectangles are helices, black arrows are beta sheets. Motifs 1–5 and important arginines are showed in coloured boxes and highlight residues that constitute the ATP-binding pocket. SK1 shares 58%, 46% and 85% amino acid identity in motifs 1–5 with DgkB, YEGs and SK2, respectively. SK1 Arg185 and Arg191 are only conserved in SK2 and CERK. Residues that were mutated in the study are represented in bold.

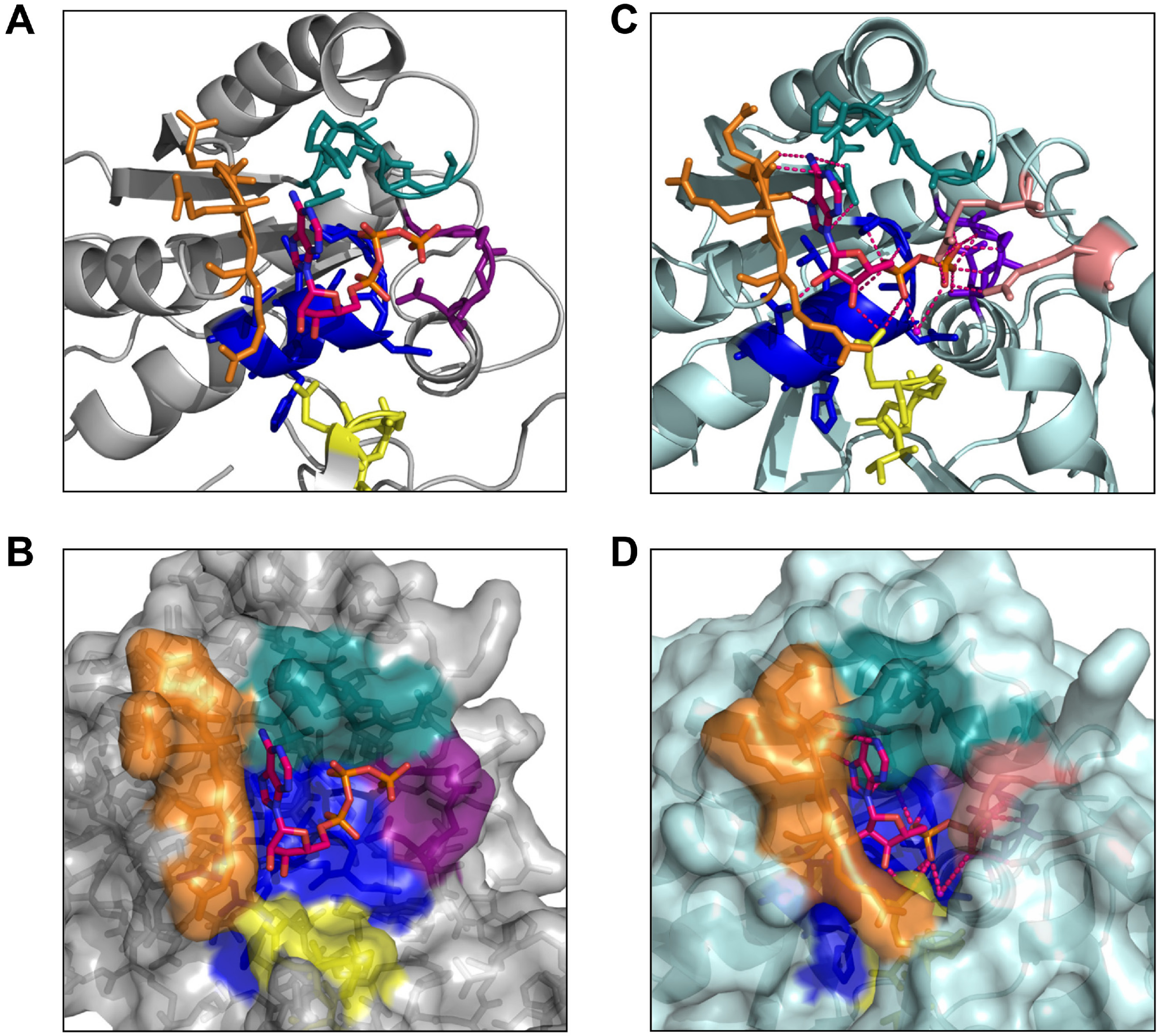

Supplementary Figure 2: Comparison of SK1 structural model with the recently solved SK1 structure. The SK1 structural model with ATP docked in ribbon **(A)** and surface view **(B)** The SK1 crystal structure co-crystallised with ADP (3VZD) in ribbon **(C)** and surface view **(D)** Each of the regions highly conserved in all SKs (see Supplementary Figure 1) that comprise the ATP-binding pocket are coloured separately; Motif 1 (^{21}LNPRGG^{26}) in teal, Motif 2 (^{54}TERR^{57}) in orange, Motif 3 (^{79}SGDGLMHE^{86}) in blue, Motif 4 (^{110}GSGN^{114}) in purple, and Motif 5 (^{340}VDGE^{343}) in yellow. Arg185 and Arg191 are coloured in light pink in the crystal structure. The atoms in ATP are coloured according to chemicals elements; oxygen in red, and nitrogen in blue. Predicted hydrogen bonding is depicted by pink dashes.

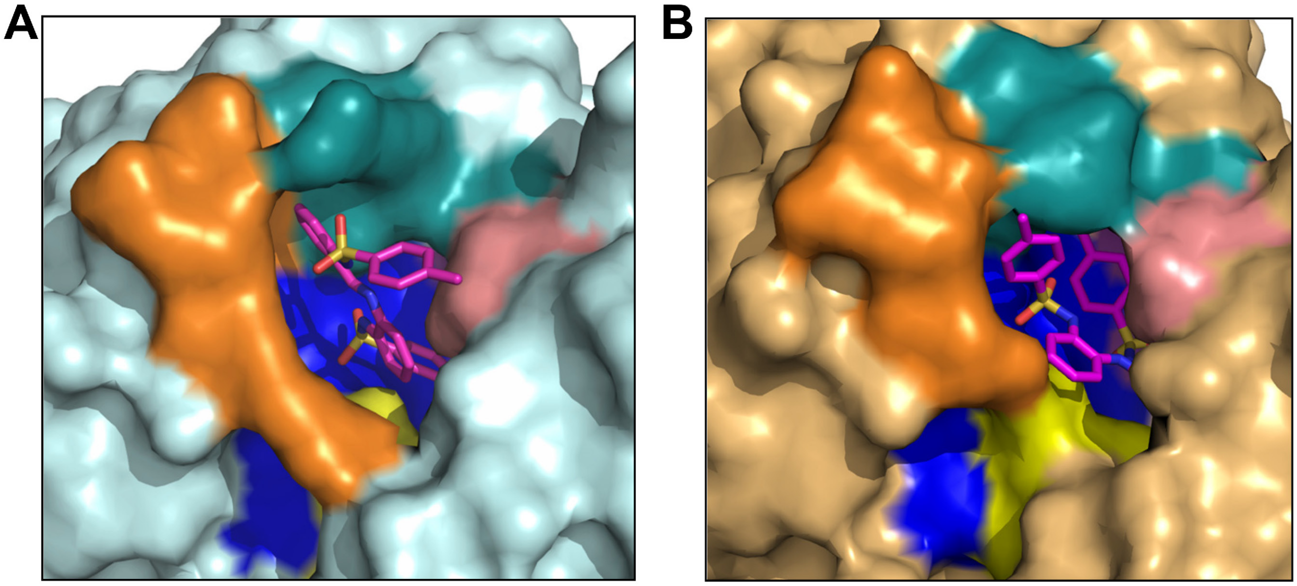

Supplementary Figure 3: Comparison of MP-A08 docking into the SK1 crystal structure and the SK2 structural model. **(A)** The SK1 crystal structure (pdbcode 3VZD) with MP-A08 docked in surface view **(B)** The SK2 model docked with MP-A08 in surface view. Each of the motifs that comprise the ATP-binding pocket are coloured using the same scheme described in Supplementary Figure 2. $SK1^{Arg185}/SK2^{Arg315}$ and $SK1^{Arg191}/SK2^{Arg321}$ are coloured in light pink in the crystal structure. The atoms in MP-A08 are coloured according to chemical elements: oxygen in red, nitrogen in blue and sulphur in yellow.

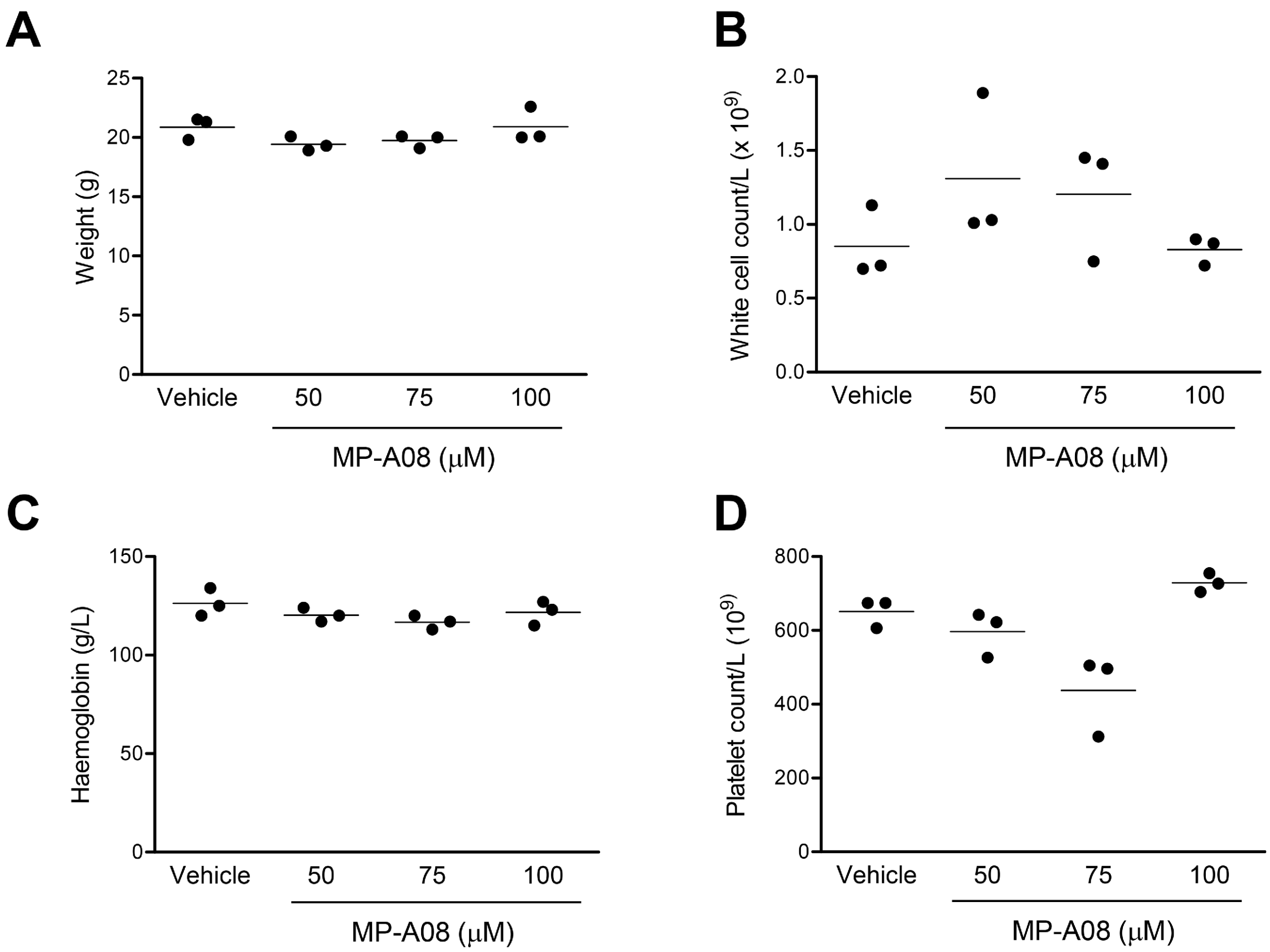

Supplementary Figure 4: Tolerance of MP-A08 in NOD/SCID mice. NOD/SCID mice were treated with 50, 75 or 100 mg/kg of MP-A08 or vehicle control (70% PEG400) by i.p. injection daily for 14 days. Animals were assessed for weight **(A)** as well as white blood cell count **(B)** blood haemoglobin **(C)** and blood platelet count **(D)**.

Supplementary Table 1: *In vitro* screening for the effects of MP-A08 against a panel of protein kinases. MP-A08 (25 µM) was tested against a panel of 140 protein kinases by the International Centre for Kinase Profiling (University of Dundee). Enzymes that MP-A08 showed a >30% increase or decrease at 25 µM were re-analysed with 250 µM MP-A08. All data represent mean ± range from duplicate determinations.

Kinase	Residual activity (% vehicle)	
	25 µM	250 µM
Abelson murine leukaemia viral oncogene homolog 1 (ABL)	103 ± 12	
AMP-activated protein kinase (AMPK)	91 ± 2	
Apoptosis Signal Regulating Kinase 1 (ASK1)	129 ± 10	
Aurora A	110 ± 10	
Aurora B	146 ± 11	63 ± 14
Breast tumour kinase (BRK)	85 ± 6	
Brain-specific kinase 1 (BRSK1)	69 ± 19	80 ± 5
BR serine/threonine kinase 2 (BRSK2)	96 ± 0	
Bruton agammaglobulinemia Tyrosine Kinase (BTK)	120 ± 15	
Calmodulin dependent kinase 1 (CAMK1)	74 ± 7	
Calmodulin dependent kinase kinase ß (CAMKKb)	76 ± 4	
Cyclin-dependent kinase 2-cyclin A complex (CDK2-Cyclin A)	104 ± 10	
Cyclin-dependent kinase 9 (CDK9-Cyclin T1)	118 ± 9	
Checkpoint kinase-1 (CHK1)	140 ± 14	129 ± 39
Checkpoint kinase-2 (CHK2)	92 ± 17	
Casein kinase 1γ2 (CK1γ2)	101 ± 9	
Casein kinase-1δ (CK1δ)	131 ± 26	76 ± 1
Casein kinase-2α (CK2)	111 ± 8	
CDC-like Kinase 2 (CLK2)	88 ± 7	
C-terminal Src kinase (CSK)	147 ± 36	84 ± 9
Death-associated protein kinase 1 (DAPK1)	122 ± 39	
Discoidin domain receptor tyrosine kinase 2 (DDR2)	101 ± 0	
Dual specificity tyrosine phosphorylation regulated kinase 1A (DYRK1A)	84 ± 4	
Dual specificity tyrosine phosphorylation regulated kinase 2 (DYRK2)	105 ± 11	
Dual specificity tyrosine phosphorylation regulated kinase 3 (DYRK3)	110 ± 12	
Elongation factor kinase (EF2K)	89 ± 6	
Eukaryotic translation initiation factor 2-alpha kinase 3 (EIF2AK3)	59 ± 6	82 ± 3
Ephrin receptor A2 (EPH-A2)	102 ± 0	
Ephrin receptor A4 (EPH-A4)	113 ± 4	
Ephrin receptor B1 (EPH-B1)	112 ± 11	
Ephrin receptor B2 (EPH-B2)	106 ± 6	
Ephrin receptor B3 (EPH-B3)	107 ± 14	
Ephrin receptor B4 (EPH-B4)	105 ± 0	

(*Continued*)

Kinase	Residual activity (% vehicle)	
	25 µM	250 µM
Extracellular signal-regulated kinase 1 (ERK1)	98 ± 10	
Extracellular signal-regulated kinase 2 (ERK2)	164 ± 24	82 ± 4
Extracellular signal-regulated kinase 7 (ERK5)	112 ± 4	
Extracellular signal-regulated kinase 8 (ERK8)	79 ± 9	
Fibroblast growth factor receptor 1 (FGF-R1)	112 ± 7	
Germinal centre kinase (GCK)	102 ± 1	
Glycogen synthase kinase-3β (GSK3β)	100 ± 0	
V-erb a erythroblastic leukaemia viral oncogene homolog 1 (HER4)	107 ± 5	
Homeodomain-interacting protein kinase 1 (HIPK1)	91 ± 10	
Homeodomain-interacting protein kinase 2 (HIPK2)	80 ± 4	
Homeodomain-interacting protein kinase 3 (HIPK3)	77 ± 2	
Insulin-like growth factor 1 receptor (IGF-1R)	60 ± 8	103 ± 3
Inhibitory κB kinase β (IKKβ)	101 ± 12	
Inhibitory κB kinase ε (IKKε)	108 ± 22	
Insulin receptor (IR)	96 ± 0	
Interleukin-1 receptor-associated kinase 1 (IRAK1)	107 ± 1	
Interleukin-1 receptor-associated kinase 4 (IRAK4)	113 ± 25	
Insulin receptor related receptor 1 (IRR)	110 ± 3	
Janus kinase 2 (JAK2)	115 ± 17	
c-Jun N-terminal kinase 1 (JNK1)	107 ± 4	
c-Jun N-terminal kinase 2 (JNK2)	111 ± 17	
c-Jun N-terminal kinase 3 (JNK3)	89 ± 18	
Lymphocyte kinase (Lck)	70 ± 13	105 ± 9
Ser/Thr kinase 11 (LKB1)	126 ± 10	
MAPK kinase kinase kinase 3 (MAP4K3)	92 ± 8	
MAPK kinase kinase kinase 5 (MAP4K5)	83 ± 1	
MAPK-activated protein kinase-2 (MAPKAP-K2)	119 ± 4	
MAPK-activated protein kinase 3 (MAPKAP-K3)	61 ± 11	70 ± 3
Microtubule-affinity-regulating kinase 1 (MARK1)	94 ± 12	
Microtubule-affinity-regulating kinase 2 (MARK2)	94 ± 8	
Microtubule affinity regulating kinase 3 (MARK3)	73 ± 3	
Microtubule-affinity-regulating kinase 4 (MARK4)	102 ± 18	
MAPK kinase kinase 1 (MAP3K1/MEKK1)	111 ± 9	
Maternal embryonic leucine zipper kinase (MELK)	131 ± 21	76 ± 15
Misshapen-like kinase 1 (MAP4K6)	124 ± 2	117 ± 7

(*Continued*)

Kinase	Residual activity (% vehicle)	
	25 µM	250 µM
MAPK kinase (MKK1)	81 ± 7	
MAPK kinase 2 (MKK2)	155 ± 4	98 ± 9
MAPK kinase 6 (MKK6)	130 ± 32	92 ± 11
Mixed lineage kinase 1 (MLK1)	90 ± 7	
Mixed lineage kinase 3 (MLK3)	97 ± 18	
MAPK interacting kinase 1 (MNK1)	119 ± 5	
MAPK interacting Kinase 2α (MNK2)	110 ± 9	
Myristoylated and palmitoylated serine/threonine protein kinase (MPSK1)	99 ± 12	
Mitogen and stress-activated protein kinase-1 (MSK1)	93 ± 10	
Mammalian sterile 20-like 2 (MST2)	106 ± 14	
Mammalian homologue Ste20-like kinase (MST3)	100 ± 6	
Mammalian sterile 20-like 4 (MST4)	119 ± 16	
NIMA related protein kinase 2a (NEK2a)	104 ± 3	
NIMA related protein kinase 6 (NEK6)	92 ± 5	
SnF1-like kinase 1 (NUAK1)	68 ± 9	91 ± 15
Oxidative stress responsive 1 (OSR1)	125 ± 2	
Stress-activated protein kinase-2α (p38α MAPK)	100 ± 6	
Stress-activated protein kinase-2ß (p38β MAPK)	118 ± 9	
Stress-activated protein kinase-4 (p38δ MAPK)	113 ± 13	
Stress-activated protein kinase-3 (p38γ MAPK)	147 ± 2	114 ± 1
p21-activated protein kinase 2 (PAK2)	94 ± 5	
p21 activated kinase 4 (PAK4)	74 ± 42	
p21 activated kinase 5 (PAK5)	105 ± 13	
p21 activated kinase 6 (PAK6)	128 ± 13	
Platelet-derived growth factor receptor (PDGFRA)	135 ± 0	74 ± 4
3-phosphoinositide-dependent protein kinase-1 (PDK1)	117 ± 6	
Phosphorylase B kinase (PhKγ1)	137 ± 4	94 ± 0
Provirus integration site for Moloney murine leukaemia virus (PIM1)	95 ± 9	
Provirus integration site for Moloney murine leukaemia virus (PIM2)	106 ± 7	
Provirus integration site for Moloney murine leukaemia virus (PIM3)	103 ± 2	
Cyclic AMP-dependent protein kinase (PKA)	100 ± 3	
Protein kinase B-α (PKBα)	95 ± 3	
Protein kinase B-β (PKBβ)	93 ± 8	
Protein kinase C-α (PKCα)	112 ± 16	
Protein kinase C-ζ (PKCζ)	139 ± 4	121 ± 29

(*Continued*)

Kinase	Residual activity (% vehicle)	
	25 µM	250 µM
Protein kinase C-γ (PKCγ)	93 ± 2	
Protein kinase D 1 (PKD1)	96 ± 10	
Polo like kinase 1 (PLK1)	107 ± 8	
p38-regulated/activated kinase (PRAK)	96 ± 2	
PKC like kinase 2 (PRK2)	127 ± 25	
Receptor interacting protein kinase 2 (RIPK2)	116 ± 5	
Rho-dependent protein kinase 2 (ROCK2)	109 ± 5	
p70 ribosomal protein S6 kinase (S6K1)	98 ± 2	
Serum and glucocorticoid-induced kinase (SGK1)	104 ± 10	
Salt inducible protein kinase 2 (SIK2)	87 ± 1	
Salt inducible protein kinase 3 (SIK3)	98 ± 9	
Smooth muscle myosin light chain kinase (SmMLCK)	76 ± 2	
Sarcoma kinase (Src)	117 ± 4	
Serine arginine protein kinase (SRPK1)	94 ± 9	
Serine/threonine kinase 33 (STK33)	151 ± 8	97 ± 4
Spleen tyrosine kinase (SYK)	100 ± 13	
Transforming growth factor-β activated kinase 1 (TAK1)	103 ± 2	
Thousand and one amino acid protein kinase (TAO1)	119 ± 2	
TANK binding kinase 1 (TBK1)	106 ± 17	
Testis-specific kinase 1 (TESK1)	106 ± 7	
Transforming growth factor-β receptor 1 (TGFβR1)	123 ± 5	
Tunica interna endothelial cell kinase (TIE2)	78 ± 8	
Tousled-like kinase 1 (TLK1)	103 ± 9	
Neurotrophic tyrosine kinase receptor, type 1 (TrkA)	91 ± 2	
Testis-specific serine kinase 1 (TSSK1)	47 ± 3	43 ± 8
Tau tubulin kinase 1 (TTBK1)	90 ± 1	
Tau tubulin kinase 2 (TTBK2)	103 ± 17	
Phosphotyrosine picked threonine kinase (TTK)	99 ± 6	
Unc-51-like kinase 1 (ULK1)	114 ± 2	
Unc-51-like kinase 2 (ULK2)	96 ± 9	
Vascular endothelial growth factor receptor 1 (VEGFR)	85 ± 7	
With no lysine deficient protein kinase 1 (WNK1)	101 ± 21	
Yamaguchi sarcoma viral oncogene homolog 1 (YES1)	106 ± 15	
Zeta chain (TCR) associated protein kinase 70 (ZAP70)	122 ± 18	

Supplementary Table 2: Primers used for generation of SK1 and SK2 mutants

Mutant		Forward Primer	Reverse Primer
SK1	N22A	5′-GTGCTGGTGCTGCTGGCGCCGCGCGGC GGCAAG-3′	5′-CTTGCCGCCGCGCGGCGCCAGCAGCA CCAGCAC-3′
	R24A	5′-GTGCTGCTGAACCCGGCCGGCGGCAAG GGCAGG-3′	5′-CCTGCCCTTGCCGCCGGCCGGGTTCAG CAGCAC-3′
	T54A	5′-CTTCACGCTGATGCTAGCTGAGCGGCG GAACC-3′	5′-GGTTCCGCCGCTCAGCTAGCATCAGCG TGAAG-3′
	E55A	5′-CGCTGATGCTCACTGCGCGCCGGAACC ACGCGCGG-3′	5′-CCGCGCGTGGTTCCGGCGCGCAGTGA GCATCAGCG-3′
	D81A	5′-GTGGTCATGTCCGGAGCCGGCCTGATG CACGAGGTG-3′	5′-CACCTCGTGCATCAGGCCGGCTCCGG ACATGACCAC-3′
	L83A	5′-CATGTCTGGAGACGGCGCCATGCACGA GGTGGTG-3′	5′-AGCCCGTTCACCACCGCATGCATCAG CCCGTCT-3′
	E86A	5′-AGACGGGCTGATGCATGCGGTGGTGAA CGGGCT-3′	5′-AGCCCGTTCACCACCGCATGCATCAG CCCGTCT-3′
	S112A	5′-AGCCTCCCAGCAGGCGCCGGCAACGC GCTGGCA-3′	5′-TGCCAGCGCGTTGCCGGCGCCTGCTGG GAGGCT-3′
	D341A	5′-GTGTGTTTGCAGTCGCCGGGGAATTGA TGGT-3′	5′-ACCATCAATTCCCCGGCGACTGCAAA CACAC-3′
	E343A	5′-GTTTGCAGTCGACGGCGCCTTGATGGT TAGCGAG-3′	5′-CTCGCTAACCATCAAGGCGCCGTCGA CTGCAAAC-3′
SK2	N152A	5′-GTTGCTTCTATTGGTGGCGCCCTTTGGG GGTCGG-3′	5′-CCGACCCCCAAAGGGCGCCACCAATA GAAGCAAC-3′
	F154A	5′-CTATTGGTCAATCCCGCGGGGGGTCGG GGCCTG-3′	5′-CAGGCCCCGACCCCCCGCGGGATTGA CCAATAG-3′
	T184A	5′-TTCAACCTCATCCAGGCAGAACGACAG AACCAC-3′	5′-GTGGTTCTGTCGTTCTGCCTGGATGAG GTTGAA-3′
	L213A	5′-GGTCTCGGGAGACGGCGCCCTCCATGA GGTGCTG-3′	5′-CAGCACCTCATGGAGGGCGCCGTCTC CCGAGACC-3′
	S242A	5′-ATCCTCCCCTGCGGCGCCGGCAACGCG CTGGCC-3′	5′-GGCCAGCGCGTTGCCGGCGCCGCAGG GGAGGAT-3′
	R315A	5′-TATCCAGAGCGAGCGATTCGCGGCCTT GGGCAGTGC-3′	5′-GCACTGCCCAAGGCCGCGAATCGCTC GCTCTGGATA-3′
	R321A	5′-TTCAGGGCCTTGGGCTCCGCGGCCTTC ACACTGGGCAC-3′	5′-GTGCCCAGTGTGAAGGCCGCGGAGCC CAAGGCCCTGAA-3′
